# Phytochemical Characterization and Antibacterial Activity of Carthamus Caeruleus L. Aqueous Extracts: In Vitro and In Silico Molecular Docking Studies

**DOI:** 10.1002/cbdv.202402662

**Published:** 2024-11-26

**Authors:** Yousra Belounis, Idir Moualek, Hillal Sebbane, Ali Dekir, Hamdi Bendif, Stefania Garzoli, Karim Houali

**Affiliations:** ^1^ Laboratory of Analytical Biochemistry & Biotechnology Research Faculty of Biological Sciences and Agricultural Sciences University Mouloud Mammeri 15000 Tizi-Ouzou Algeria; ^2^ Laboratory of Applied Organic Chemistry Synthesis of Biomolecules and Molecular Modelling Group Sciences Faculty Chemistry Department Badji-Mokhtar Annaba University Box 12 23000 Annaba Algeria; ^3^ Department of Natural and Life Sciences Faculty of Sciences University of M'sila, University Pole Laboratory of Ethnobotany and Natural Substances ENS Algers Road Bordj Bou Arreiridj 28000 M'sila Algeria; ^4^ Department of Chemistry and Technologies of Drug Sapienza University P. le Aldo Moro, 5 00185 Rome Italy

**Keywords:** Antibacterial activity, Aqueous extracts, HPLC, Molecular docking

## Abstract

In order to valorize natural resources and the traditional use of medicinal plants in Algeria, this study exploits the antibacterial effect of *Carthamus caeruleus* L. Since there are few studies on this plant despite its notable therapeutic potential, this work aims to characterize the chemical composition of *Carthamus caeruleus* L. leaf and root aqueous extracts and to evaluate their antibacterial activity through an *in vitro* and *in silico* studies. Spectrophotometric assays and HPLC results revealed 22 components in the roots and 16 in the leaves. Disc diffusion and microdilution methods were used to study the antibacterial properties against nine standard bacterial strains. The results showed that roots exhibited the best activity on most tested strains. Both extracts were also able to inhibit the growth of *Staphylococcus aureus* ATCC 25923 and *Escherichia coli* ATSC 25922. Furthermore, no nucleic acid leakage or membrane damage was detected. However, molecular docking of the molecules indicates that some constituents have significant affinity and stability for DNA gyrase. Gallic acid, luteolin, myricetin, and orientin were found to have the highest score. The molecular docking data suggest, for the first time, that the antibacterial activity may be caused by the inhibition of DNA gyrase.

## Graphical abstract

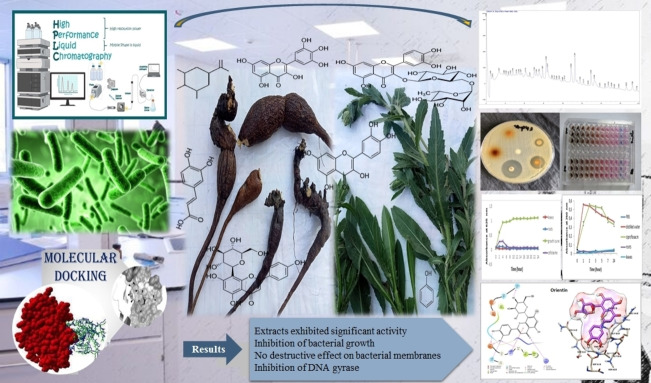

## Introduction

Bacterial infections represent one of the major problems affecting public health. Every year, thousands of people die from acute respiratory infections, enteric infections, tuberculosis, etc.^[1]^ Until the discovery of antibiotics in 1928, medicinal plants represented the only way to fight bacteria and were used in the treatment of infectious diseases.^[2]^ Today, several countries as well as the WHO (World Health Organization) have invested considerable effort in exploiting the therapeutic effects of plant‐based remedies.^[3]^

Due to the increase and emergence of bacterial resistance to antibiotics and the decline in the number of new antibiotics in recent years,^[4]^ exploiting the antibacterial potential of plant extracts represents a promising approach. In addition to possessing several mechanisms of action, compared to antibiotics, which reduce the risk of developing resistance,^[5]^ the long‐standing use of plants seems to be more accepted by patients as a source of antimicrobial compounds.^[2]^ Moreover, given the unwanted side effects of antibiotics significantly higher than those due to natural compounds, the use of phytochemicals has more advantages.^[6]^

Medicinal plants have known to have a complex composition of secondary metabolites with remarkable antimicrobial properties, such as flavonoids, alkaloids, and phenolic acids.[^7^, ^8^] However, many compounds possess antimicrobial activity, but few studies have reported their mechanisms of action. Thus, understanding the mechanism of action and the relationship between the structure and the activity of these phytochemical compounds is essential to ensure optimal and effective use in the development of antimicrobial drugs.[^2^, ^9^] Currently, several studies have been based on the antibacterial mechanism of plants and the structure‐activity relationship.[^2^, ^10^]

In Algeria, *Carthamus caeruleus* L. is a medicinal plant with great therapeutic potential. Its roots are used in traditional medicine in the treatment of various skin problems, including burns.[^11^, ^12^] Furthermore, since skin infections affect the healing process of injured skin,^[13]^ it will be interesting to investigate the antibacterial potential of *C. caeruleus* L. to facilitate wound healing. To the best of our knowledge, very few studies have revealed the antibacterial potential of this plant, and those conducted on leaf and root aqueous extracts are even scarcer.

The aim of this study is to characterize the chemical composition and evaluate *in vitro* the antibacterial potential of *C. caeruleus* L. root and leaf aqueous extract. In addition, this work aims to increase our knowledge and understanding of the antibacterial potential of the investigated extracts, focusing on the possible modes of action regarding disruption of membrane integrity and the structure‐activity relationship through an *in silico* study. A molecular docking study was also carried out to identify a probable target for the extract‘s bioactive components in order to justify its antimicrobial potential.

## Results and Discussion

### Phytochemical Analysis

Polyphenols are extremely diverse bioactive molecules that include flavonoids and tannins. These molecules have received the most research attention.^[14]^ In this study, the determination of polyphenols, flavonoids, and condensed tannin content of *C. caeruleus* L. aqueous extracts (leaves and roots) was carried out and summarized in Table 1.

**Table 1:** Polyphenol, flavonoid, and condensed tannin contents of *C. caeruleus* L. aqueous extracts.

Dosage	Polyphenols mg GAE/g extract	Flavonoids mg QE/g extract	Condensed tannins mg TAE/g extract
Leaves	83.54±4.69^[a]^	4.41±0.048^[a]^	62.95±0.5^[a]^
Roots	21.19±0.37^[b]^	0.72±0.013^[b]^	27,28±1.04^[b]^

Values represent the mean±SEM of three separate experiments using triplicate samples in each (n=3) for both leaf and root extracts. Different superscript letters in the same column indicate significant differences (p<0.05). GAE: gallic acid equivalents; QE, quercetin equivalents; TAE: tannic acid equivalents.

The present study showed that leaves were richer in polyphenols, flavonoids, and condensed tannins (83.54±4.69 mg GAE/g, 4.41±0,048 mg QE/g, 62,95±0,5 mg TAE/g of dry extract, respectively) compared to roots aqueous extract (21.19±0.37 mg GAE/g, 0.72±0.013 mg QE/g, 27.28±1.04 mg TAE/g, respectively) (Table 1). This variation aligns with the findings of Habibou et al. (2019)^[15]^ and Pistelli et al. (2019),^[16]^ who claimed that this difference is explained by the fact that different organs of the same plant can have different amounts of polyphenols. This is related to a number of factors, like abiotic stressand in particular the aerial parts undergo more stress than other parts of the plant. Comparing our results to those of Baghiani et al. (2009)^[17]^ and Ouda et al. (2021),^[18]^ they found lower amounts of polyphenols in the root methanolic extract of the same plant (12.966±0.727 and 13.08±0.22 mg GAE/g dry extract, respectively). Interestingly, their studies indicated higher flavonoid content in the root extracts (2,231±0.146 and 5.02±0.55 mg QE/g extract, respectively) compared to our findings. This difference could be related to the polarity of solvents used in the preparation of extracts.^[19]^

Notably, our study stands out as the first to quantify the polyphenol and flavonoid contents in *C. caeruleus* leaves aqueous extract, as well as to measure condensed tannins in both leaf and root extracts. This gap in the existing literature highlights the novelty of our findings.

### HPLC Profile

Table 2 provides a detailed summary of the HPLC profiles obtained from the analyses of the extracts. The components in both extracts were identified by comparing their retention times and peak areas with those of standard molecules. This study represents the first characterization of *C. caeruleus* L. root and leaf aqueous extracts using HPLC, revealing a significant abundance of active compounds. Specifically, 22 molecules were identified in the roots and 16 in the leaves

**Table 2:** Molecules identified by HPLC in *C. caeruleus* L. aqueous extracts, classification and their antibacterial activities.

Class	Components identified/Reference of the classification	Leaf extract	Root extract	Inhibition of bacterial strains/Reference
		Area (%)	Area (%)	
Phenolic acids	2,3‐dimethyl cinnamicacid^[20]^	ND	1.5895	ND
	3,4,5‐trimethoxybenzoic acid^[20]^	ND	2.1882	ND
	Dihydroxycinnamic acid^[20]^	ND	2.1875	ND
	Caffeic acid^[21]^	12.458	ND	*B. cereus*, *P. aeruginosa*, *S. aureus*, *E. coli* ^[22]^ *L. monocytogenes* ^[21]^
	Ferulic acid^[20]^	ND	2.8991	*P. aeruginosa* ATCC 10145, *L. monocytogenes* ATCC 15313, *S. aureus* CECT 976, *E. coli* CECT 434^[20]^
	Gallic acid^[20]^	0.8401	ND	*P. aeruginosa* ATCC 10145, *S. aureus* CECT^[20]^
	Hydroxycinnamic acid^[20]^	4.5507	ND	ND
Isovanillic acid^[23]^	ND	4.1639	ND	Isovanillic acid^[23]^
m‐Anisic acid^[24]^	ND	0.9167	ND	m‐Anisic acid^[24]^
*p*‐Coumaric acid^[20]^	3.1140	2.0014	*L. monocytogene*, *E. coli* ^[25]^, *B. subtilis*, *Salmonella typhimurium* ^[26]^	*p*‐Coumaric acid^[20]^
Rosmarinic acid^[21]^	ND	3.0362	*E. coli*, *B. subtilis*, *S. aureus*, *Pseudomonas* spp, *L. monocytogenes* ^[21]^	Rosmarinic acid^[21]^
Tannic acid^[27]^	2.4420	2.4771	*E. coli*, *S. aureus* ^[28]^	Tannic acid^[27]^
Flavonoids				
Flavonols	Myricetin^[29]^	2.0132	1.2076	*S.aureus*, *P. aeroginosa*, *K. pneumonia*, *E. coli* ^[30]^
	Quercetin^[31]^	1.8188	1.6805	*S. aureus* ^[32]^, *E. coli*, *P. aeroginosa* ^[31]^
	Rutin^[33]^	ND	2.5702	*E. coli*, *Klebsielasp*, *P. aeroginosa*, *B. subtilis* ^[32]^
Flavanones	Hesperidin^[34]^	ND	0.4340	*S. aureus*, *E. coli*, *P. auruginosa*, *B.cereus* ^[34]^
	Naringenin 7 glucoside^[35]^	3.0687	ND	ND
Flavones	Luteolin^[36]^	1.0471	1.2398	*S. aureus*, *L. monocytogenes* ^[37]^
	Orientin^[38]^	4.4491	4.9447	ND
	Vitexin^[39]^	ND	2.9931	*E. coli*, *P. auruginosa* ^[39]^
Other	1,2‐dihydroxybenzene	16.551	ND	ND
	Alkaloid: Caffeine^[40]^	ND	1.5039	*S. aureus*, *K. pneumonia*, *E. coli*, *B. subtilis* ^[40]^
	Coumarin	0.9569	1.4114	ND
	Hydroxy‐quinone	3.1823	3.3714	ND
	*p*‐hydroxybenzaldehyde	7.4644	2.7577	*S. aureus*, *B. cereus*, *S. Typhimurium* ^[41]^
	Resorcinol	12.058	1.4332	ND
	Vanillin	2.2794	4.5950	*E. coli*, *Listeria inocula* ^[42]^

ND: not defined

According to Table 2, most of the identified molecules can be classified into several groups: phenolic acids, flavonoids (flavonols, flavanones, and flavones), and alkaloids. It should be noted that, according to the literature, most of them showed antibacterial activity when tested alone, according to the references cited in the table.

Among the phenolic compounds, HPLC analysis detected molecules that are known to have potent antibacterial activity (Table 2). For example, caffeic acid, which, according to Kępa et al. (2018),^[43]^ is one of the most potent and promising antimicrobial agents. We also mention quercetin^[6]^ and rutin^[7]^ against a wide range of pathogens and quinone derivatives, which belong to the drug molecules with potent antibacterial activity and used in several pharmaceutical applications.^[44]^

### Antibacterial Activity

Medicinal plants are known to treat a variety of diseases due to their antimicrobial properties.^[45]^ They represent an important source of new drugs.^[46]^ In this context, the present study examined the antibacterial activity of *C. caeruleus* L, determined its efficacy on bacterial growth, and screened its mechanism of action. The results of the inhibition zones and MICs of leaf and root aqueous extracts are summarized in Table 3.

**Table 3:** Antibacterial activity of *Carthamus caeruleus* L. leaves and roots.

Strains	Disc diffusion method (DIZ)			MICs (mg/mL)	
	Leaves (0.3 g/mL)	Roots (0.6 g/mL)	Antibiotics	Leaves (0.3 g/mL)	Roots (0.6 g/mL)
Bacillus cereus ATCC 10876	6^[a]^	6^[a]^	26.5±0.7^[b]^	ND	23.0±0.012^[c]^
Enterococcus faecalis ATCC 29212	6^[a]^	10.5±0.7^[b]^	25.5±0.7^[c]^	30.0±0.012^[a]^	2.34 ±0.02^[a]^
Enterococcus faecalis ATCC 49452	6^[a]^	10.5±0.7^[b]^	25.5±0.7^[c]^	37.5^[a]^	4.69^[b]^
Escherichia coli ATCC 25922	6^[a]^	11.5±0.7^[b]^	29.5±0.7^[c]^	150.0±0.062^[a]^	6.25±0.02^[b]^
Klebsiella pneumonia ATCC 700603	6^[a]^	6^[a]^	29.0±1.4^[b]^	150.0±0.002^[a]^	50.0±0.021^[b]^
Listeria monocytogenes ATCC 15313	6^[a]^	6^[a]^	23.5±0.7^[b]^	75.0±0.002^[a]^	15.75^[b]^
Pseudomonas aeruginosa ATCC 27853	6^[a]^	10.5±0.7^[b]^	31.0±1.4^[c]^	37.5^[a]^	150.0±0.052^[b]^
Staphylococcus aureus ATCC 25923	6^[a]^	14.5±0.7^[b]^	29.5±0.7^[c]^	50.0±0.02^[a]^	50.0±0.021^[a]^
Staphylococcus aureus ATCC 43300	6^[a]^	16.5±0.12^[b]^	26.0±1.4^[c]^	75.0^[a]^	75.0^[a]^

Values represent the mean±SEM of three separate experiments using triplicate samples in each (n=3) for both leaf and root extracts. Different superscript letters in the same row indicate significant differences (*p*<0.05). DIZ: diameter of inhibition zone, MICs: minimum inhibitory concentrations, ND: not defined.

#### Disc Diffusion Method

**Figure 1:**
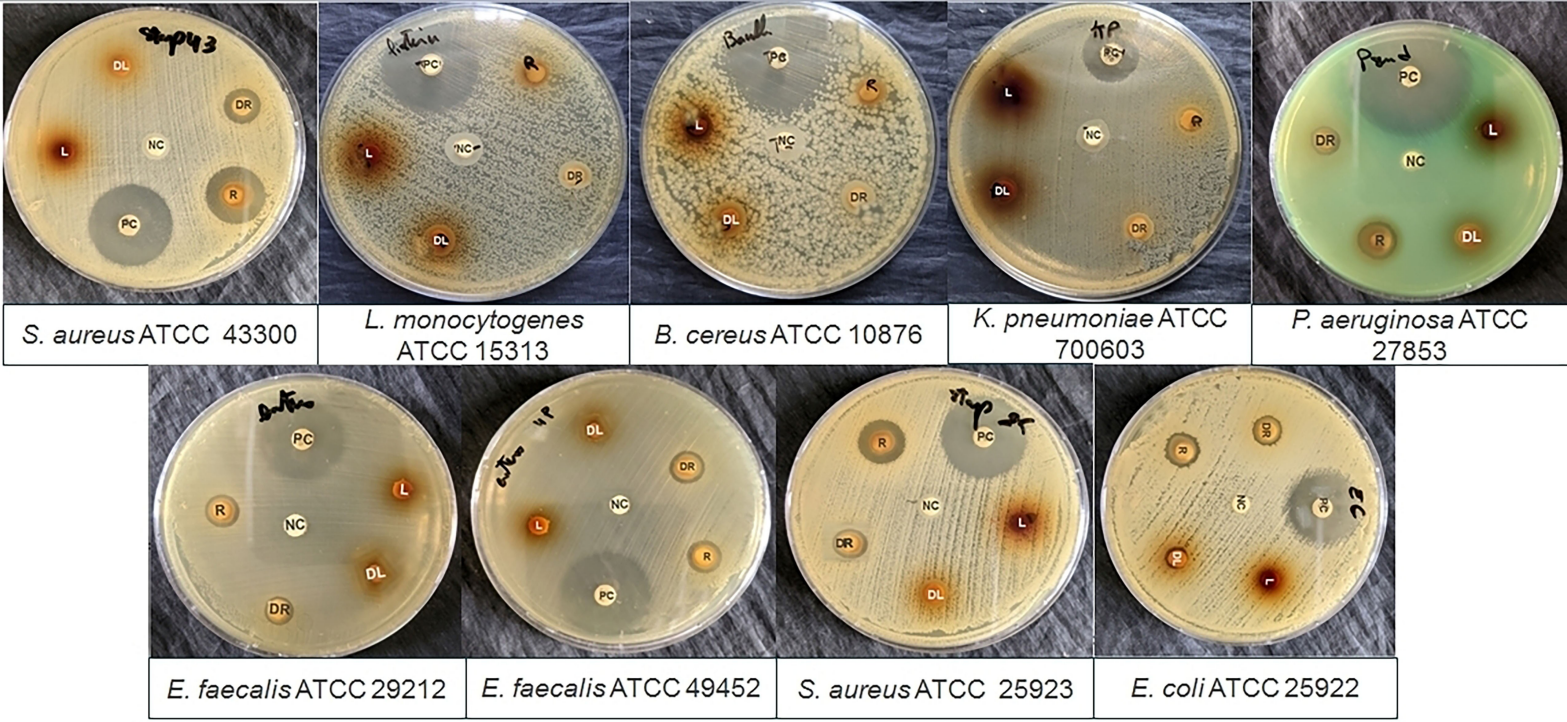
Inhibition zones obtained for *Carthamus caeruleus* l. aqueous extracts against various bacterial strains tested. PN: Positive control, NC: Negative control, R: Root extract, DR: Dilution (1 : 2) of root extract, L: Leaf extract, DL: Dilution (1 : 2) of leaf extract.

The disk diffusion method was used to evaluate the antibacterial activity of *C. caeruleus* L. Table 3 and Figure 1 summarizes the inhibition zones obtained from this test. Discs impregnated with aqueous root extract showed significant inhibition of bacterial growth, with inhibition zones ranging from 10.5±0.7 mm to 16.5±0.12 mm for sensitive strains such as *E. faecalis* ATCC 49452, *E. faecalis* ATCC 29212, *E. coli* ATCC 25922, and *S. aureus* ATCC 25923, as well as against antibiotic‐resistant strains, as defined by the CLSI (M100, 2020), including *S. aureus* ATCC 43300 (methicillin‐resistant, MRSA) and *P. aeruginosa* ATCC 27853 (a producer of β‐lactamases). The best activity was recorded against *S. aureus* ATCC 43300 for root aqueous extract (16.5±0.12 mm). However, no inhibition was recorded against *B. cereus* ATCC 10876, *K. pneumonia* ATCC 700603 (Extended‐Spectrum Beta‐Lactamase, ESBL), or *L. monocytogenes* ATCC 15313. Leaves showed no activity against any of the tested bacterial strain. The zones of inhibition of the tested antibiotics were over 23 mm.

According to Kimathi et al. (2022),^[47]^ the antibacterial potential of plant extracts can be classified depending on the diameter of the inhibition zone. Thus, the diameters indicate low antimicrobial activity for an inhibition zone between 6 and 9 mm, moderate for an inhibition zone between 9 and 12 mm, high for an inhibition zone between 13 and 16 mm, very high for an inhibition zone between 16 and 19 mm, and remarkable when this exceeds 20 mm. Based on this evaluation, root extract showed very high activity against *S. aureus* ATCC 43300 (16.5±0.12 mm), high activity against *S. aureus* ATCC 25923 (14.5±0.7 mm), and moderate activity against *P. aeruginosa* ATCC 27853 (10.5±0.7 mm), *E. coli* ATCC 25922 (11.5±0.7 mm), *E. faecalis* ATCC 29212, and *E. faecalis* ATCC 49452 (10.5±0.7 mm).

The antibacterial efficacy of *C. caeruleus* methanolic leaf and root extracts was also confirmed by Saffidine et al. (2013).^[11]^ Roots with an inhibition zone of 20 mm showed high activity against *S. aureus* ATCC 25923 and *B. cereus* ATCC 10876. On the other hand, for strains such as *E. coli* ATCC 25922, *K. pneumonia* ATCC 700603 and clinical strains (*Proteus spp* and *P. aeruginosa*) the extract studied showed no activity. For leaf methanolic extract, Saffidine et al. (2013)^[11]^ obtained an inhibition zone of 20 mm for *S. aureus* ATCC 25923 and 10 mm for *P. aeruginosa* and *B. cereus* ATCC 10876. But it was inactive against *E. coli* ATCC 25922, *K. pneumonia*, and *Proteus spp* clinical strain.

The present study revealed that root extract possessed better antibacterial activity than leaves (*p*≤0.05). Saffidine et al. (2013)^[11]^ came to the same conclusion, indicating that roots have better activity than leaves against the bacteria tested. This supports the results of ethnobotanical surveys, where roots are the only plant parts used in traditional.[^48^, ^49^] Furthermore, the difference in antibacterial activity between the two plant parts tested can be attributed to the difference in phytochemical compounds present in each extract.^[47]^

#### Minimum inhibitory concentrations (MICs)

**Figure 2:**
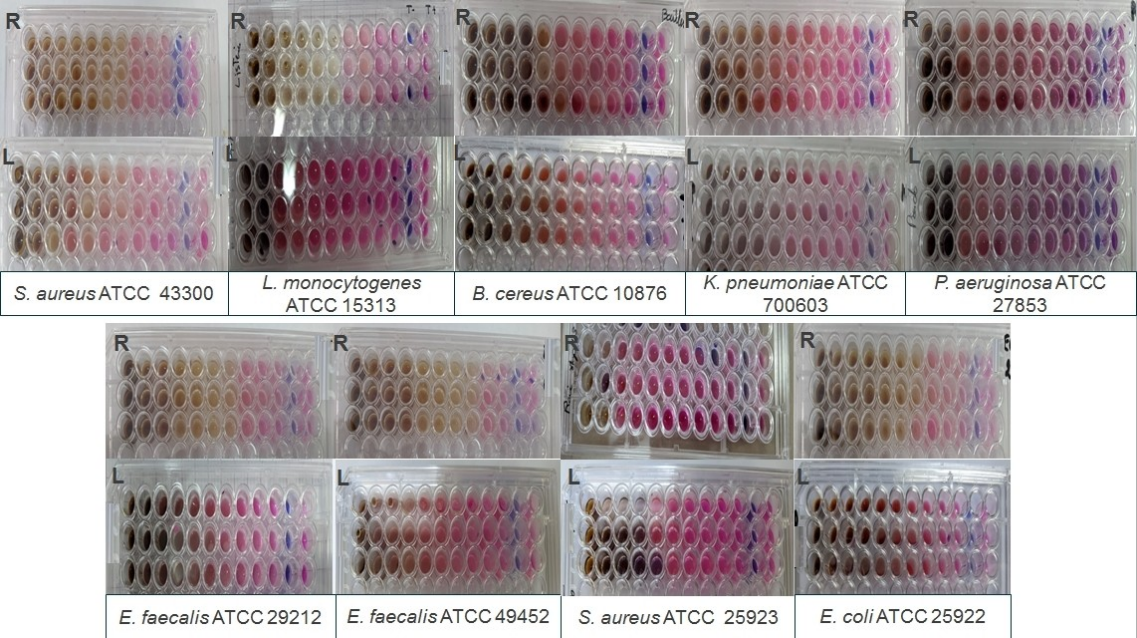
MIC of *Carthamus caeruleus* L. aqueous extracts against various bacterial strains tested. R: Root extract, L: Leaf extract.

A microdilution test was used in this study to assess the antibacterial activity of the two extracts against the used strains. The results are expressed as minimum inhibitory concentrations (MIC) and are summarized in Table 3 and Figure 2. Root extract showed the best activity on most of the strains tested, with MICs ranging from 2.34±0.02 to 150±0.052 mg/mL, except for the two staphylococcus strains, which had similar activity to leaf extract, and *P. aeruginosa*, where leaf showed the best activity (*p*≤0.05).

The leaves recorded MICs between 30±0.012 and 150±0.062 mg/mL, while this extract revealed no bacterial inhibition in agar diffusion tests. According to Wijenayake et al. (2022),^[50]^ this difference can be attributed to the physical properties of a drug, which can affect its antibacterial activity. Thus, the lack of antibacterial activity of leaves when using a solid medium may be related to the limited diffusion of the extract. Furthermore, Wijenayake et al. (2022)^[50]^ suggest that drug molecules disperse easily in water (a liquid medium), enabling better contact with the bacterial target than with a solid medium, therby ensuring greater efficacy. Górniak et al. (2019)^[2]^ also revealed that the low diffusion of some flavonoids in agar gives poor results in the agar diffusion test despite their known antimicrobial activity.

Saffidine et al. (2013)^[11]^ tested the MICs of *C. caeruleus* leaf and root methanolic extracts, where they recorded better activity compared to our study, with values of 1.3 mg/mL for *S. aureus* with root aqueous fraction and values between 0.12 and 0.68 mg/mL against *B. cereus* for root organic fractions. For leaf aqueous fraction, Saffidine et al. (2013)^[11]^ recorded an MIC of 12.5 mg/mL on *S. aureus*. According to Górniak et al. (2019),^[2]^ this difference may result from the specific properties of the solvent used for extraction, as various solvents can selectively dissolve different compounds, thereby influencing the overall composition and efficacy of the extract. Górniak et al. (2019)^[2]^ also suggested that methanolic extracts generally possess the highest antimicrobial activity due to the extraction of large numbers and high concentrations of flavonoids. Furthermore, according to Borges et al. (2013),^[20]^ this discrepancy in the obtained results can be explained by the difference in the method used to determine MICs.

#### Effect of Extracts on Growth Curve

Unlike traditional counting methods for surveying bacterial growth, measurement of suspension turbidity is faster, non‐destructive, and inexpensive.^[51]^ This method allows to assess the effectiveness of an extract by measuring its turbidity for 24 hours. According to Wang et al. (2020),^[51]^ testing the antibacterial efficacy of an extract over a long period of time is very important for a drug.

**Figure 3:**
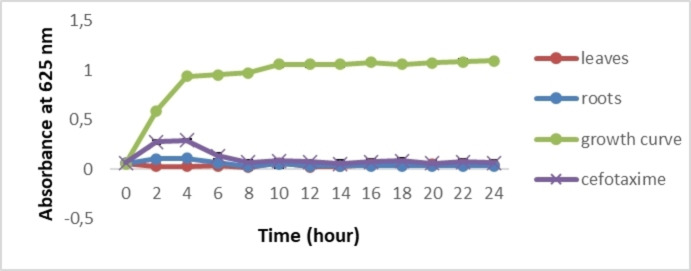
Growth curve of *E. coli* treated with *C. caeruleus* L. extracts.

**Figure 4:**
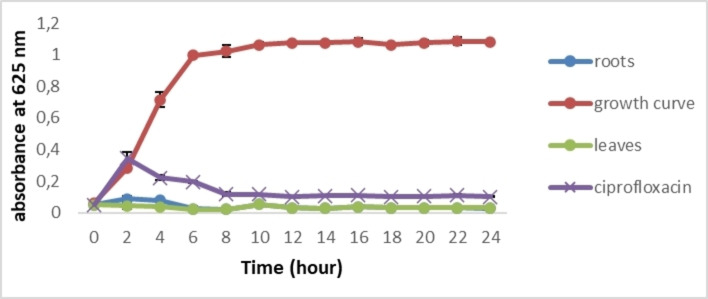
Growth curve of *S. aureus* treated with *C. caeruleus* L. extracts.

The effects of leaf and root extracts at concentrations of 0.2 g/mL and 0.075 g/mL, respectively, were tested on *S. aureus* and *E. coli* growth (Figures 3 and 4). In comparison with the bacterial growth curve, a total growth inhibition of the two selected bacteria was recorded. After 24 hours, a significant difference was observed between the extracts or antibiotics and the growth curve (*p*≤0.05). These results show, for the first time, that *C. caeruleus* is effective in inhibiting bacterial growth

Similar results were obtained by Wang et al. (2020),^[51]^ when they tested the efficacy of monocaprins against four bacterial strains (*S. aureus*, *E. coli*, *P. aeruginosa*, and *B. subtilis*). Bacterial growth inhibition can be attributed to the presence of phytochemicals in the extract, which, according to Veiko et al. (2023),^[35]^ are able to inhibit *S. aureus* and *E. coli* growth. In addition, they reported that polyphenols modify the activity of genes responsible for metabolic control and genetic information, decrease ribosome activity, alter nucleic acid synthesis, and inhibit bacterial topoisomerases. Górniak et al. (2019)^[2]^ have also shown that the inhibition of bacterial growth can be related to the stabilization of the topoisomerase II–DNA cleavage complex, which leads to altering cell division and/or inhibiting chromosome replication.

#### Determination of Cell Membrane Permeability: Release of Nucleic Acids

The first step towards the optimal use of medicinal plant extracts as natural antimicrobial agents is to understand their mechanism of action.^[52]^ For this, assessing nucleic acid leakage provides information on the state of the bacterial membrane and its integrity. Indeed, numerous studies have demonstrated that leakage of cytoplasmic material can indicate significant and irreparable damage. Phenolic compounds are known to increase membrane permeability and cause leakage of cellular components such as ions, proteins, and nucleic acids.^[20]^

**Figure 5:**
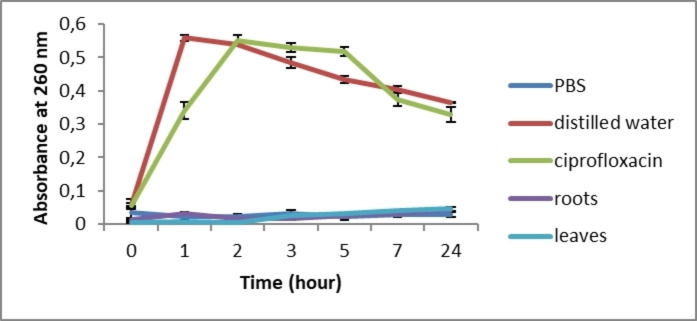
Nucleic acid leakage from *E. coli* treated with *C. caeruleus* L. extracts.

**Figure 6:**
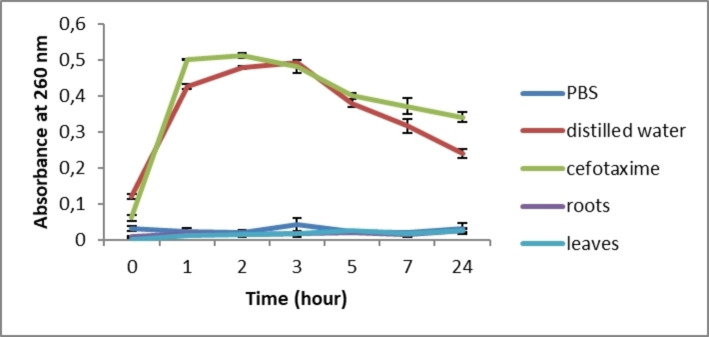
Nucleic acid leakage from *S.aureus* treated with *C. caeruleus* L. extracts.

After treatment with *C. caeruleus* L. aqueous extracts, no increase in absorbance at 260 nm was recorded for the two treated bacteria, *S. aureus* and *E. coli*, compared to distilled water and antibiotics (*p*≤0.05) (Figures 5 and 6). The extracts, therefore, showed no significant difference from the suspension tested in PBS. These data suggest, for the first time, that *C. caeruleus* extracts does not damage bacterial membranes, since no membrane lysis or nucleic acid leakage was observed.

Ekom et al. (2021)^[53]^ revealed a significant increase in nucleic acid absorbance after treatment of three bacterial strains (*P. aeruginosa*, *S. aureus*, and *E. coli*) with *Capsicum annuum* extract. These results indicated that the bacterial membrane was damaged, allowing nucleic acids to be released. This study confirmed that this alteration of the membrane justifies the bactericidal effect of the extract.

In summary, the present study revealed the antibacterial potential of *C. caeruleus* against nine standard strains (both gram‐positive and gram‐negative). According to Adamczak et al. (2019),^[54]^ it is necessary to use standardized methods to compare the antibacterial activity of an extract on the same bacterial strains with other researches. Thus, our study followed CLSI guidelines, which standardized all factors that can lead to variable results, such as strain sensitivity, the type of broth or agar used, and the size of the bacterial inoculum.^[2]^

Gram‐negative bacteria are known to be more resistant than Gram‐positive ones due to the presence of an outer membrane.[^8^, ^55^] However, in this study, the extract was effective simultaneously on both Gram‐negative and Gram‐positive bacteria. This shows that the nature of the membranes of the two bacterial types does not influence the activity of the *C. caeruleus* extracts.

The reported antibacterial activity of *C. caeruleus* can be attributed to the presence of secondary metabolites such as polyphenols, flavonoids, alkaloids, tannins, phenolic acids, and coumarins.^[54]^ The mechanism of action of these compounds is not yet fully elucidated; however, their action involves numerous sites in the cell.^[56]^ For example, myricetin inhibits DnaB helicase, DNA gyrase, and E. coli DNA and RNA polymerases, which play a role in DNA replication and elongation.^[57]^ Ibrahim et al. (2020)^[58]^ revealed that caffeic acid inhibits bacterial RNA polymerase. Furthermore, Ragunathan & Ravi, (2015)^[32]^ suggested that quercetin and rutin inhibit isoleucyl‐tRNA‐synthetase (one of the proteins crucial to protein synthesis) and dihydrofolate reductase (an enzyme important for folic acid synthesis) in *S. aureus*, respectively.

Several studies have examined the structure‐activity relationship of phytochemicals to explain their antibacterial mechanisms.^[56]^ According to Sánchez‐Maldonado et al. (2011)^[10]^ and Papuc et al. (2017),^[59]^ this activity is linked to the number and position of hydroxyl (OH) groups. Adamczak et al. (2019)^[54]^ suggested that the presence of OH groups in the A ring is essential for the antibacterial activity of flavones, particularly at positions C‐5, C‐6, and C‐7. However, Shamsudin et al. (2022)^[60]^ revealed that flavones are more active against Gram‐positive bacteria than flavanones, due to the presence of a C2=C3 double bond in flavones, which is absent in flavanones. In addition, the hydrophobic character of flavonols seems to be important in their mode of action. These hydrophobic molecules easily penetrate phospholipid membranes and, once inside, exert their antibacterial activity^.[61]^

Previous research has suggested a close relationship between the antioxidant and antibacterial activities of drugs. We know that iron is an essential element for bacterial survival and growth. The antibacterial activity of polyphenols can therefore be attributed to their ability to chelate iron.^[59]^ The results of Papuc et al. (2017)^[59]^ supported this proposal, where, after they added three pure hydrolysable tannins to a bacterial suspension and observed inhibition of the tested bacteria, incorporating iron into the culture medium restored bacterial growth. Thus, they suggested that the antibacterial activity of these tannins is linked to their ability to chelate iron. In addition, Jubair et al. (2021)^[46]^ showed a relationship between oxidative stress and antibiotic resistance and suggested that a drug with both antioxidant and antibacterial activity is preferable as an alternative drug.

Finally, other studies have also mentioned that the antibacterial activity of an extract may be the result of synergy between its different phytochemical compounds.^[54]^ Sharma et al. (2013)^[7]^ supported this hypothesis, revealing a synergistic effect between rutin and other flavonoids such as quercetin, morin, kaempherol, myricetin, and fisetin. Moreover, according to Semwal et al. (2016),^[30]^ additive or synergistic interactions enhanced the activity of myricetin associated with other active ingredients.

### Molecular Docking Study

To validate our experimental findings and elucidate the mechanism of action of the extract components, we conducted docking simulations on various biological targets. Notably, our investigation revealed that these biomolecules exhibited robust affinity and stability towards DNA gyrase. Consequently, we undertook a detailed molecular docking study specifically focusing on the DNA gyrase enzyme.

**Figure 7:**
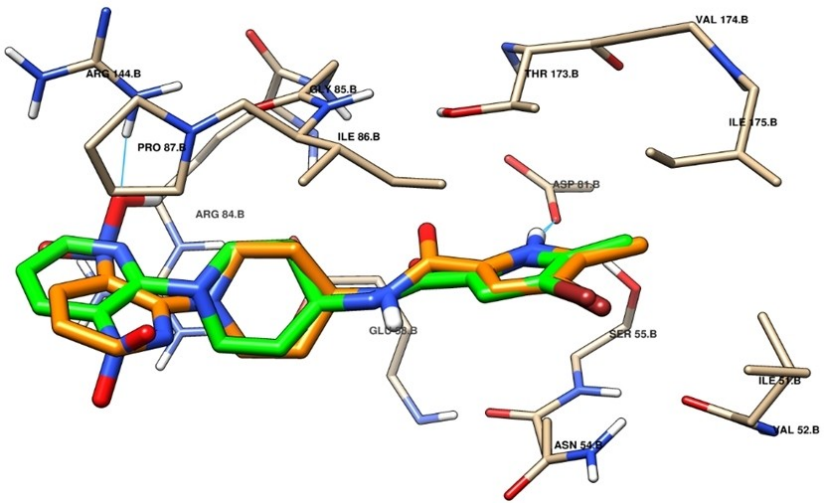
There‐docking of the co‐crystallized ligand (PDB: 3U2D), hydrogen bonding interactions are depicted by blue lines.

DNA gyrase, an enzyme present in bacteria and some archaea, holds a pivotal role in DNA replication, transcription, and repair by being a part of the topoisomerase enzyme class, which is responsible for manipulating DNA structure. Several plant secondary metabolites serve as catalytic inhibitors of DNA gyrase, primarily functioning through competitive inhibition of GyrB ATPase activity.^[62]^ Leveraging this mechanism of action, we conducted molecular docking of our biomolecules to probe their stability, selectivity, and interactions with DNA gyrase. To validate our docking protocol, we commenced with re‐docking the co‐crystallized ligand, achieving successful results with an RMSD below 1 Å (Figure 7).

**Figure 8:**
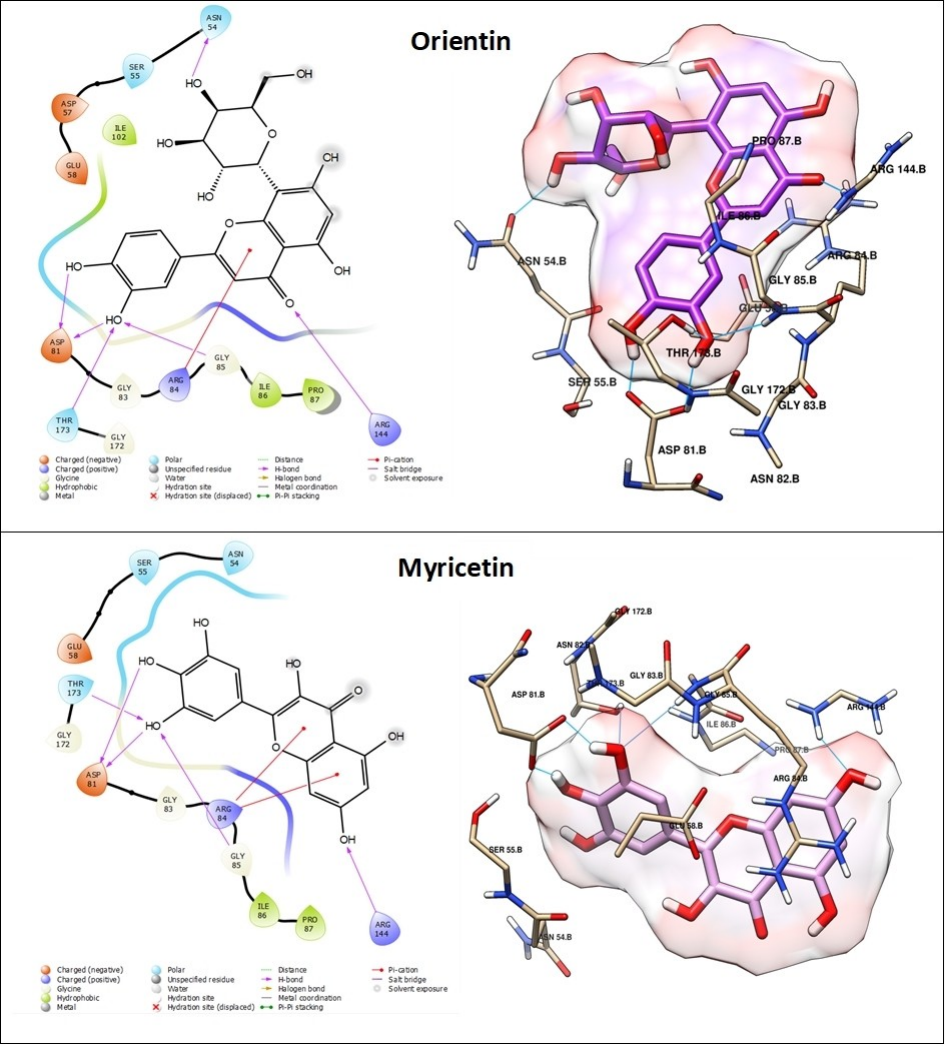
3D and 2D of the Luteolin and Gallic acid into DNA gyrase (PDB: 3U2D). Hydrogen bonding interactions shown in cyan line.

**Figure 9:**
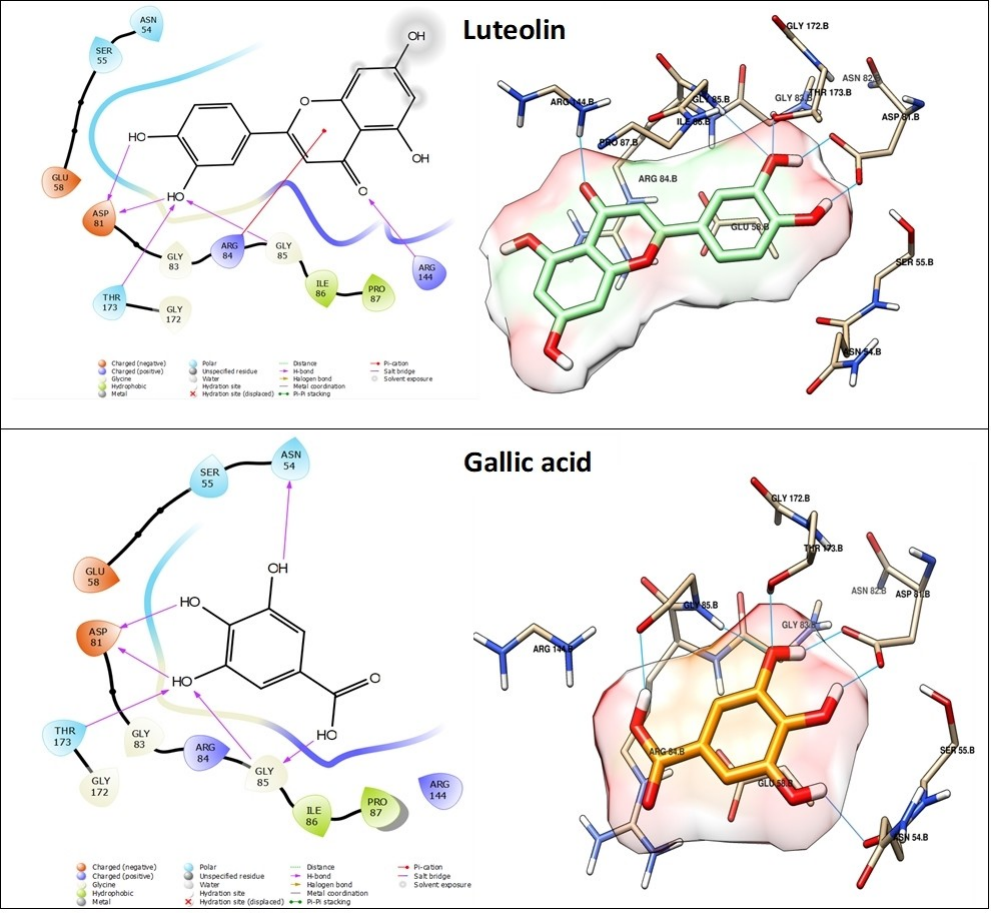
3D and 2D of the Luteolin and Gallic acid into DNA gyrase (PDB: 3U2D). Hydrogen bonding interactions shown in cyan line.

The assessment of molecular docking outcomes demonstrated that all docked components exhibited considerable stability when contrasted with the co‐crystallized ligand, a fact supported by their respective docking score values (Table 4). Figures 8 and 9 showcase the overlay of some docked components within the DNA gyrase cavity. We have thoroughly documented and elucidated the mode of interaction for the top four molecules such as Gallic acid, Luteolin, Myricetin and Orientin within DNA gyrase, relying on their respective docking score values.

**Table 4:** Ranked of docked derivatives after docking study.

Compound code	Docking Score (kcal/mol)	Compound code	Docking Score (kcal/mol)
Ferulic acid	−5.794	Quercetin	−5.674
Isovannilic acid	−5.705	Hesperidin	−5.057
m‐anisic acid	−5.561	Hydroxycinnamic acid	−5.168
Rosmarinic acid	−5.443	dihydroxycinnamic acid	−5.486
Caffeic acid	−5.889	Hydroxy‐quinone	−5.264
Caffeine	−5.484	Naringenine‐7‐Glucoside	−5.991
**Gallic acid**	−**6.327**	*p*‐coumaric acid	−5.564
**Luteolin**	−**6.057**	Resorcinol	−5.125
**Myricetin**	−**6.155**	Vanillin	−4.253
**Orientin**	−**6.389**	Vitexin	−5.369
Rutin	−5.868	Co‐cristalized ligand	−5.470

Orientin exhibited the most promising result and holds the potential to function as a potent inhibitor of DNA gyrase. This claim is substantiated by its exceptional docking score of −6.389 kcal/mol, surpassing that of the other compounds analyzed in the study (refer to Table 4). This compound engages in a total of six hydrogen bond interactions and one pi‐cation interaction. Specifically, its hydroxyl groups form five hydrogen bonds with ASN 54, ASP 81, THR 173, and GLY 85 residues, while an additional hydrogen bond is established via the oxygen atom of the carbonyl group. Moreover, a pi‐cation interaction occurs between the heterocycle and ARG 144 (Figure 8). Next, Myricetin exhibits a favorable docking score of −6.155 kcal/mol (Table 4) and is involved in a total of five hydrogen bond interactions and two pi‐cation interactions. More specifically, its hydroxyl groups form five hydrogen bonds with ASP 81, THR 173, and ARG 144. Furthermore, two pi‐cation interactions are observed between its cycles and ARG 84 (Figure 8). However, Luteolin demonstrates similar interactions to Myricetin, forming five hydrogen bonds with ASP 81, THR 173, and ARG 144. However, it differs in forming one pi‐cation interaction with ARG 84 residue instead of two, as depicted in Figure 7. In the case of Gallic acid, it achieves a notable docking score of −6.327 kcal/mol (Table 4), attributed to its formation of six hydrogen bonds with ASN 54, ASP 81, THR 173, and GLY 85 facilitated by its four hydroxyl groups (Figure 9). The stability and affinity observed in the compounds are attributed to the substantial number of hydrogen interactions, along with hydrophobic interactions involving heterocycles and phenolic cycles. These findings, derived from the docking study against DNA gyrase, corroborate the biological results effectively.

Our molecular docking results are supported by Górniak et al. (2019),^[2]^ who showed that flavonoid hydroxyl groups enable better interactions with gyrase compared with methoxy groups. They reported that quercetin inhibits E. coli DNA gyrase through its 5, 7, and 3′OH groups, which bind to the B subunit of gyrase.

## Conclusions

In this study the antibacterial activity of *C. caeruleus* leaf and root aqueous extracts against nine standard bacteria strains was evaluated. The diameter of the inhibition zone and the MIC results indicate that the antibacterial activity of roots is higher than that of leaves. A significant inhibition of selected bacterial growth by both extracts was recorded. However, although the extracts revealed no effect on bacterial membrane integrity, molecular docking results indicate that this antibacterial potential may be due to inhibition of DNA gyrase.These findings support the traditional use of *C. caeruleus* in the treatment of wounds and prevention of bacterial infections; nevertheless, a thorough study against other microorganisms and othermodes of action is required to explore potential applications of these extracts in the pharmacological industry.

## Experimental Section

### Chemicals and Instruments

The chemicals and solvents used in this study were purchased from Sigma‐Aldrich. The solvents were of HPLC grade (purity ≥99.9 %), and the reagents were commercially available, analytically and chemically pure (purity ≥98 %), used without any special treatment, unless otherwise stated. Absorbances were measured using a MEDLINE MD 2000 UV‐visible spectrophotometer.

### Plant Collection

Roots and leaves of *C. caeruleus* L. were collected in May 2022 at Freha, Tizi‐Ouzou, Algeria (36° 45′ 8.42′′N; 4° 18′ 55.80′′E). Botanical identification was carried out by Dr. Benghanem Nabil, botanist at the National Superior School of Agronomy (ENSA), Algiers, Algeria. A sample was placed in the herbarium of the Mouloud Mammeri University (UMMTO) under N° 2023/UMMTO/21. The plant was cleaned, dried, and ground to a fine powder using an electric grinder, then stored for later use.

### Aqueous Extraction

Aqueous extraction was prepared in distilled water (10 %, w/v) and the mixture was magnetically agitated at room temperature for 24 hours. The macerate underwent two stages of filtration: first, it was filtered through a sieve, and then it was filtered using Whathman paper No1. After that, the filtrate was lyophilized and stored in the dark.

### Phytochemical Study

#### Determination of Total Polyphenol Content

Total polyphenol content was determined according to the method described by Singleton & Rossi (1965)^[63]^ using the Folin‐Ciocalteu reagent. The principle of this technique is based on the oxidation of polyphenols by phosphomolybdic and phosphotungstic acids (which constitute the “Folin‐Ciocalteu” reagent). Once the folin is reduced, a blue color appears, and the absorbance was measured at 760 nm.^[64]^ 200 μL of extract (1 mg/mL) were mixed with 1 mL of Folin‐Ciocalteu reagent diluted 1/10 (v/v) and freshly prepared. The mixture was incubated in the dark for 4 minutes at room temperature, then 800 μL of sodium carbonate (Na_2_CO_3_) solution (7.5 %, w/v) were added. After shaking and incubation under the same conditions for 45 minutes, the absorbance was measured at 760 nm (MEDLINE MD 2000 UV‐visible spectrophotometer), and the results were expressed as milligrams of gallic acid equivalent per gram of dry extract (mg GAE/g) using a gallic acid curve (10 to 100 μg/mL).

### Total Flavonoid Content (TFC)

The quantification of total flavonoid content was carried out according to Akrout et al. (2011)^[65]^ using aluminum trichloride (AlCl_3_) which forms a complex with flavonoids.^[19]^ In summary, 1 mL of the extract (10 mg/mL) was combined with 1 mL of aluminum trichloride methanolic solution (2 %), and then incubated for 15 min at room temperature. The absorbance was then measured at 430 nm against a blank in which the extract was replaced by distilled water, and the results were expressed in milligrams of quercetin equivalent per gram of dry extract (mg QE/g).

#### Condensed Tannins

The determination of condensed tannins was estimated using Folin‐Denis according to the method described by Hmid et al., (2016).^[66]^ For this test, 100 μL of sample (10 mg/ml) were mixed with 750 μL of distilled water, 1 mLof Folin‐Denis reagent, and 2 mL of a saturated Na_2_CO_3_ solution. The mixture was incubated in the dark for 30 minutes at room temperature, the absorbance was read at 760 nm, and the results were expressed in milligrams of tannic acid equivalent per gram of dry extract (mg TAE/g).

#### High‐Performance Liquid Chromatography (HPLC) Analysis

The RP‐HPLC‐DAD system was used for the separation and characterization of polyphenolic compounds in *C. caeruleus* L. extracts using an AGILENT 1100 series system coupled to a diode array detector (DAD G1315B), using different wavelengths depending on the studied molecules. The separation was effected on a C18 reversed‐phase column (dimensions 4.6×250 mm, particle diameter 5 micrometers). The mobile phase contained two eluents: acidified water (0.2 % acetic acid, pH 3.1) and acetonitrile. The flow rate was maintained at 1.5 mL/min for 30 minutes at room temperature, starting with 95 % H_2_O and ending with 100 % acetonitrile. The solutions of the extracts were prepared in methanol at a concentration of 100 mg/mL. The phytochemical compounds that constitute the extract were identified by comparing their retention times with those of the peaks of individual reference substances (cinnamic acid, di‐cinnamic acid, sinapic acid, ferrulic acid, gallic acid, *p*‐benzoic acid, *n*‐cinnamic acid, resorcylic acid, salicylic acid, syringic acid, aesculetin, apigenine, glu‐7‐luteonine, hydroxy‐quinone, *p*‐benzaldehyde, vanilline, quercetin, 2,5‐dimethylhydroxycinnamic acid, 3,4,5‐trimethoxy‐trans‐cinnamic acid, 3,4,5‐trimethoxybenzoic acid, cafeic acid, isovanillic acid, *O*‐anisic acid, *m*‐anisic acid, synergic acid, tannic acid, *trans*‐cinnamic acid, *trans*‐dimethoxycinnamic acid, 3‐hydroxy‐4‐methoxycinnamic acid, caffeine, hesperidine, isoramenitine, luteoline, naringenine7‐glucoside).[^67^, ^68^] The chromatograms were reported in Supplementary Materials.

### Bacterial Strains

The antibacterial activity of *Carthamus caeruleus* L. aqueous extracts was evaluated on nine standard bacterial strains obtained from the American Type Culture Collection (ATCC) *Staphylococcus aureus* ATCC 25923, *Staphylococcus aureus* ATCC 43300, *Escherichia coli* ATCC 25922, *Klebsiella pneumonia* ATCC 700603, *Pseudomonas aeruginosa* ATCC 27853, *Bacillus cereus* ATCC 10876, *Enterococcus faecalis* ATCC 49452, *Enterococcus faecalis* ATCC 29212, *Listeria monocytogenes* ATCC 15313.

### Disc Diffusion Method

This method follows the protocol described by Kimathi et al. (2022).^[47]^ First, bacteria were revived in Brain Heart Infusion Broth (BHIB) medium (Oxoid). Then, using a 24‐hour strain, a standardized suspension was prepared at an optical density (OD) of 0.08 to 0.1 (at 625 nm) according to the recommendations of the Clinical and Laboratory Standards Institute (CLSI, 2020). Mueller Hinton medium plates were inoculated with this suspension, and then sterile blank discs were loaded with 20 μL of aqueous extracts at a concentration of 0.3 and 0.6 g/mL for leaves and roots, respectively. Negative control discs (impregnated with distilled water) and positive control disks (antibiotics) were used. The plates were incubated for 24 hours at 37 °C, and the inhibition zones were measured in millimeters (mm).

### Minimum Inhibitory Concentrations (MIC)

The microdilution method is used to determine the minimum inhibitory concentrations (MICs) of the two extracts for the bacterial strains already selected, as described by Kępa et al. (2018),^[43]^ following the recommendations of CLSI, (2020). Using sterile 96‐well plates, a series of dilutions were prepared in a Mueller‐Hinton broth (MHB) (Oxoid) from concentrations of 0.3 and 0.6 g/mL for leaves and roots, respectively, to obtain a final volume of 50 μL in each well. Standardized bacterial inoculum was prepared at an absorbance of 0.1 (625 nm, 3.8×10^8^ CFU/mL), then diluted to the hundredth in MHB (5×10^5^ CFU/mL), where CFU was the number of colony‐forming units. 50 μL of the standardized suspension was added to each well except the negative growth control to make a final volume of 100 μL. Plates were homogenized and incubated for 20 hours at 37 °C.

After incubation, bacterial growth was revealed using resazurin. 25 μL of resazurin (at 0.02 % w/v) were added to each well, and then the plates were incubated for two hours at 37 °C. The color change from violet to pink was used to assess bacterial growth. The minimum inhibitory concentration is the lowest concentration of extract that inhibits bacterial growth after incubation, with no color change in resazurin (which remains purple).^[8]^

The 11th and 12th wells were tested as negative growth control (MHB only) and positive growth control (bacterial inoculum only), respectively. The results were repeated in triplicate.

### Effect of Extracts on Growth Curve

Growth curves were Growth curves were established to assess the extracts’ efficacy in inhibiting the growth of two bacterial strains, one Gram‐positive and one Gram‐negative (*Staphylococcus aureus* ATCC 25923, and *Escherichia coli* ATCC 25922), using the method described by Wang et al. (2020).^[51]^

From a standardized bacterial suspension (OD of 0.1 at 625 nm) prepared in BHIB (Oxoid) from a culture in the logarithmic growth phase (18 hours), a growth curve of the two strains was first plotted. Briefly, one volume of suspension and one volume of BHIB were mixed in a sterile flask, and then incubated at 37 °C. Cell growth was followed by sampling every two hours and measuring absorbance at 625 nm. For the extract‘s effect on bacterial growth, a sterile flask containing one volume of suspension and one volume of extract at a concentrations of 0.2 g/mL and 0.075 g/mL (for leaves and roots, respectively) was prepared, following the same steps as above.

Reference antibiotics selected according to CLSI (Ciprofloxacin for *S. aureus* and Cefotaxime for *E. coli*) were used as a positive control for growth inhibition. The effect of the extract or antibiotics on growth was tested in triplicate on the same day under the same conditions.

### Determination of Cell Membrane Permeability: Release of Nucleic Acids

To investigate the mechanism of action of *C. caeruleus*, nucleic acid leakage was used as an indicator of severe cytoplasmic membrane damage, as described by Ekom et al. (2021).^[53]^ Two strains (*S. aureus and E. coli*) were cultured in Mueller‐Hinton broth for 18 hours. After standardization to an absorbance of 0.5 and centrifugation at 3000 rpm for 15 minutes, the pellet was washed three times with phosphate‐buffered saline (PBS) (10 mM, pH 7.4), and then suspended in the same buffer.

The test consisted of transferring 0.5 mL of the previously prepared suspension and 0.5 mL of the extracts (0.2 g/mL) into sterile eppendorfs. After incubation at 4 °C, at each different time interval, the eppendorf was centrifuged at 10,000 rpm at 4 °C, and the supernatant was transferred to further eppendorfs. The quantity of nucleic acids in the supernatant was measured according to Ehrt & Schnappinger (2003).^[69]^ After adding absolute ethanol to the supernatant, tubes were incubated for 20 minutes at −4 °C to precipitate nucleic acids, and then centrifuged a second time at the same speed. The resulting pellet was suspended in PBS, and the absorbance was measured at 260 nm.

The bacterial suspension incubated with distilled water and antibiotics (Ciprofloxacin for *S. aureus* and Cefotaxime for *E. coli*) served as a positive control for nucleic acid release. In parallel, the bacterial suspension incubated in physiological water was used as a negative control. The test was performed in triplicate.

### Molecular Docking

The molecular docking study was conducted at the Laboratory of Applied Organic Chemistry, Synthesis of Biomolecules and Molecular Modelling Group, Sciences Faculty, Chemistry Department, Badji‐Mokhtar Annaba University:

### Preparation of Protein

The protein structure of DNA gyrase, identified by the code 3U2D,^[70]^ was acquired from the Protein Data Bank and loaded into the Maestro 20.3 workspace. Using the Protein Preparation Wizard in the Schrödinger platform, the protein underwent meticulous preprocessing steps. These included assigning bond orders, removing water molecules, and adjusting protonation states to accommodate a pH range of 7.0±2.0. Subsequently, hydrogen bonds were incorporated, and ions were removed to optimize the structure. Refinement of the hydrogen bond network using PROPKA in the “Refine” tab ensured further structural accuracy, while eliminating water molecules with fewer than three hydrogen bonds to non‐water molecules enhanced the overall quality. The structure was then subjected to energy minimization using the OPLS3 force field, with an RMSD (Root‐mean‐square deviation) threshold of 0.30 Å,^[71]^ refining the geometry and alleviating any steric hindrances.

### Preparation of Ligands

The compounds were prepared using the Ligprep module within Maestro 20.3. Utilizing the OPLS3 force field, the preparation process ensured consistency by setting the pH to 7.0±2.0. The output format remained in the default setting as used by Maestro, ensuring compatibility and ease of further analysis.^[72]^

### Receptor Grid Generation

The receptor grid file was generated using the receptor grid generation panel, defining key active sites essential for the glide ligand docking simulation. The ligand‐binding site was determined either by the co‐crystallized ligand within the workspace or through the sitemap module in the Schrödinger platform.^[73]^

### Molecular Docking Analysis

The compounds were prepared and subsequently docked into the active sites of the DNA gyrase protein using standard precision docking. Before this, the co‐crystallized ligand was docked into the protein‘s active site to predict the docking score and molecular interactions, offering crucial information about the binding process.^[74]^

### StatisticalAnalysis

All tests were conducted in three independent tests, and expressed as mean±standard error of mean (SEM). Analysis of these results was performed with one‐way analysis of variance ANOVA followed by a Post‐hoc test (Tukey and Tamhan) using SPSS software (version 25), and results were considered statistically significant at *p*<0.05.

## Funding

This research did not receive any specific grant from funding agencies in the public, commercial, or not‐for‐profit sectors.

## Author Contributions

Yousra Belounis: Data curation, Investigation, Methodology, Validation, Formal analysis. Idir Moualek: Data curation, Investigation, Methodology, Validation, Formal analysis. Hillal Sebbane: Data curation, Methodology, Validation. Ali Dekir: Data curation, Investigation, Methodology, Validation, Formal analysis. Hamdi Bendif: Conceptualization, Investigation, Data curation. Stefania Garzoli: Data curation, Investigation, Writing‐review & editing. Karim Houali: Writing‐original draft, Project administration, Supervision

## Conflict of Interests

The authors declare no conflict of interest.

1

## Supporting information

As a service to our authors and readers, this journal provides supporting information supplied by the authors. Such materials are peer reviewed and may be re‐organized for online delivery, but are not copy‐edited or typeset. Technical support issues arising from supporting information (other than missing files) should be addressed to the authors.

Supporting Information

## Data Availability

Data will be made available on request.
