# Supplementary material for: Phytochemical Characterization and Antibacterial Activity of Carthamus Caeruleus L. Aqueous Extracts: In Vitro and In Silico Molecular Docking Studies
Source: Chem Biodivers. 2024 Nov 26;22(1):e202402662. doi: 10.1002/cbdv.202402662 (PMC11741163; doi:10.1002/cbdv.202402662)
Supplement: Supplementary file 1 — Supporting Information [file CBDV-22-e202402662-s001.pdf]

Chemistry & Biodiversity

Supporting Information

Phytochemical Characterization and Antibacterial Activity of Carthamus Caeruleus L. Aqueous Extracts: In Vitro and In Silico Molecular Docking Studies

Yousra Belounis, Idir Moualek, Hillal Sebbane, Ali Dekir, Hamdi Bendif,* Stefania Garzoli,* and Karim Houali

SUPPLEMENTARY MATERIALS

The chromatograms obtained in this study are presented in the Figures S1, S2, S3 and S4 for the leaf extract, and in the Figures S5, S6, S7, S8, and S9 for the root extract, compared to the chromatogram of reference standards (figure S10).

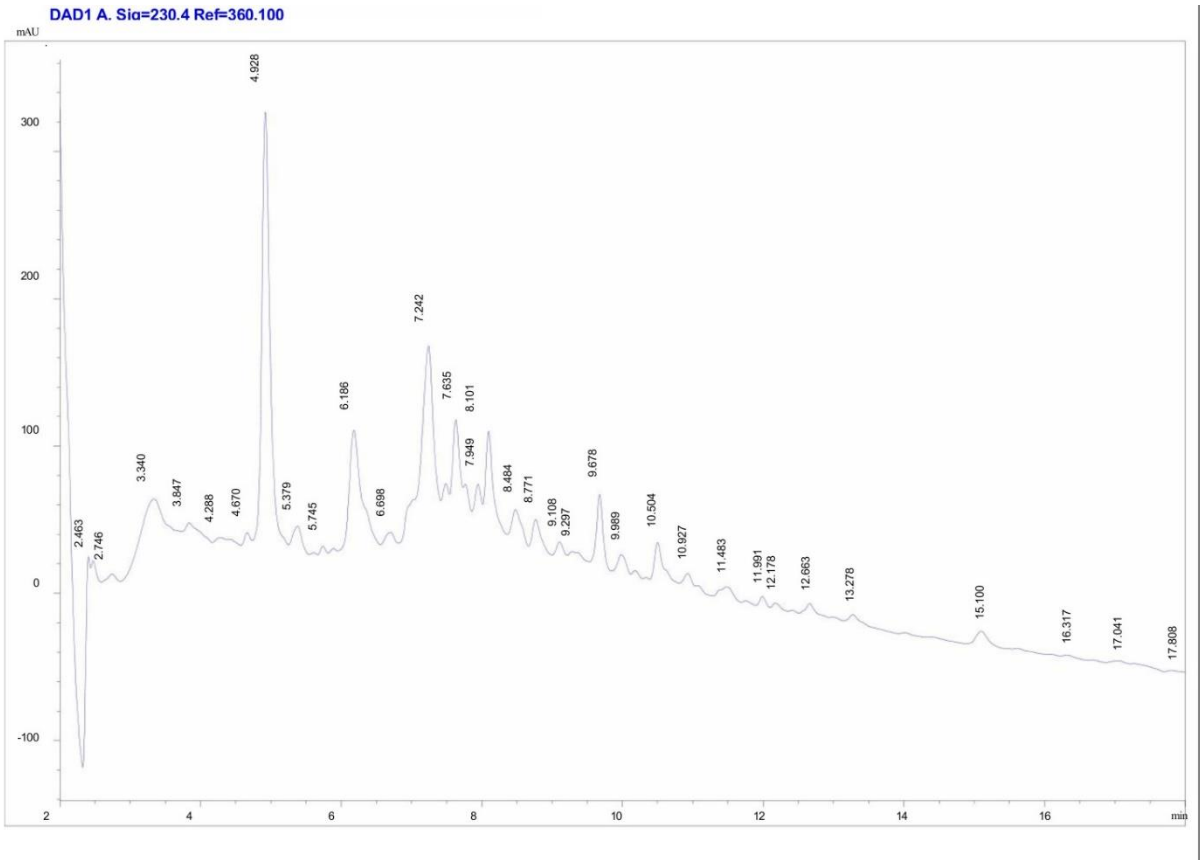

Figure S1. Chromatogram of *C. caeruleus* L. leaf aqueous extract at 230.4 nm

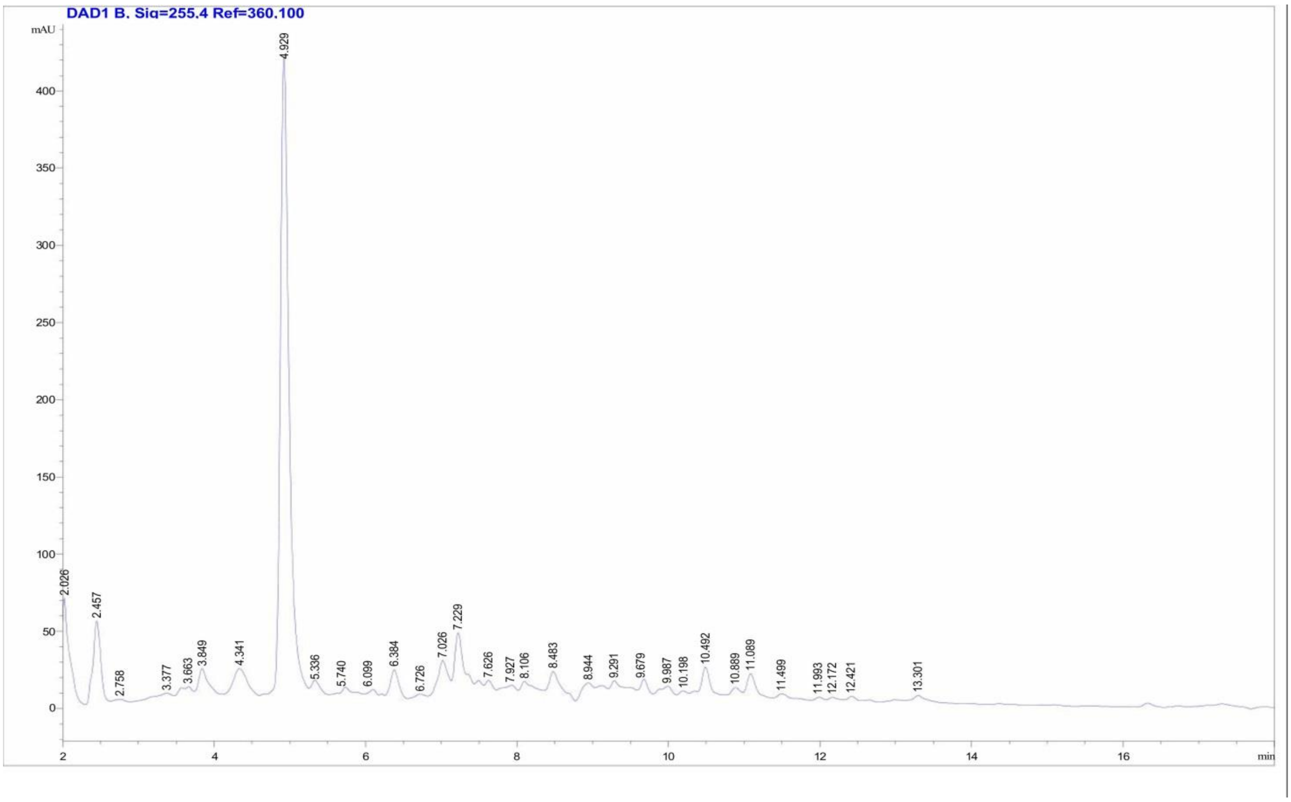

Figure S2. Chromatogram of *C. caeruleus* L. leaf aqueous extract at 255.4 nm

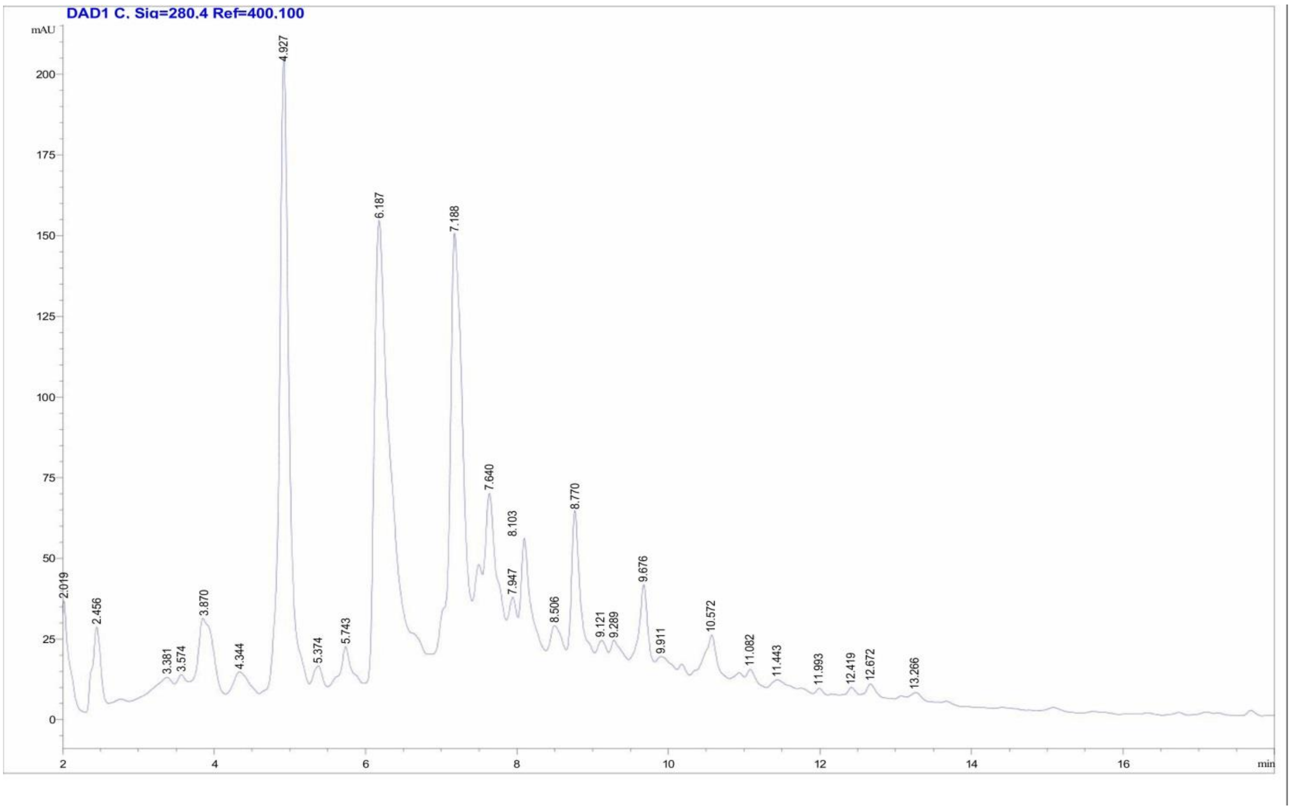

Figure S3. Chromatogram of *C. caeruleus* L. leaf aqueous extract at 280.4 nm

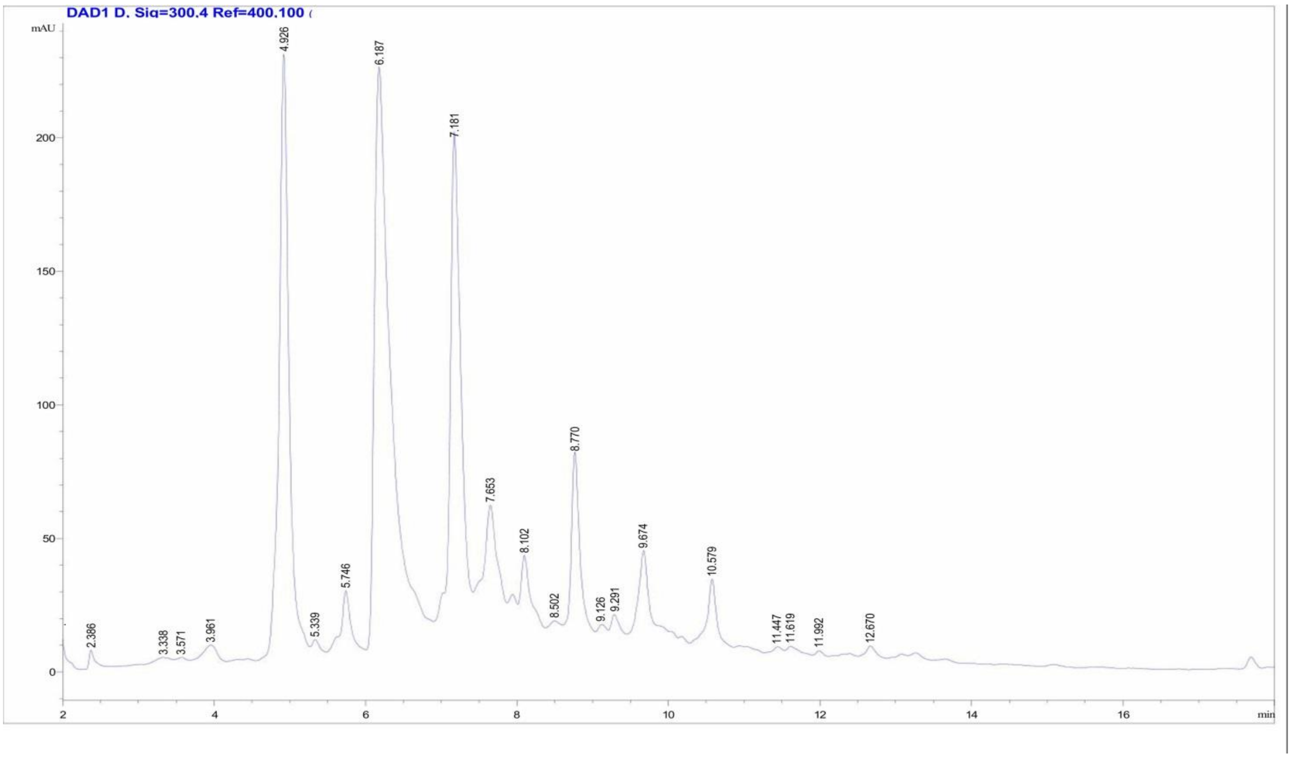

Figure S4. Chromatogram of *C. caeruleus* L. leaf aqueous extract at 300.4 nm

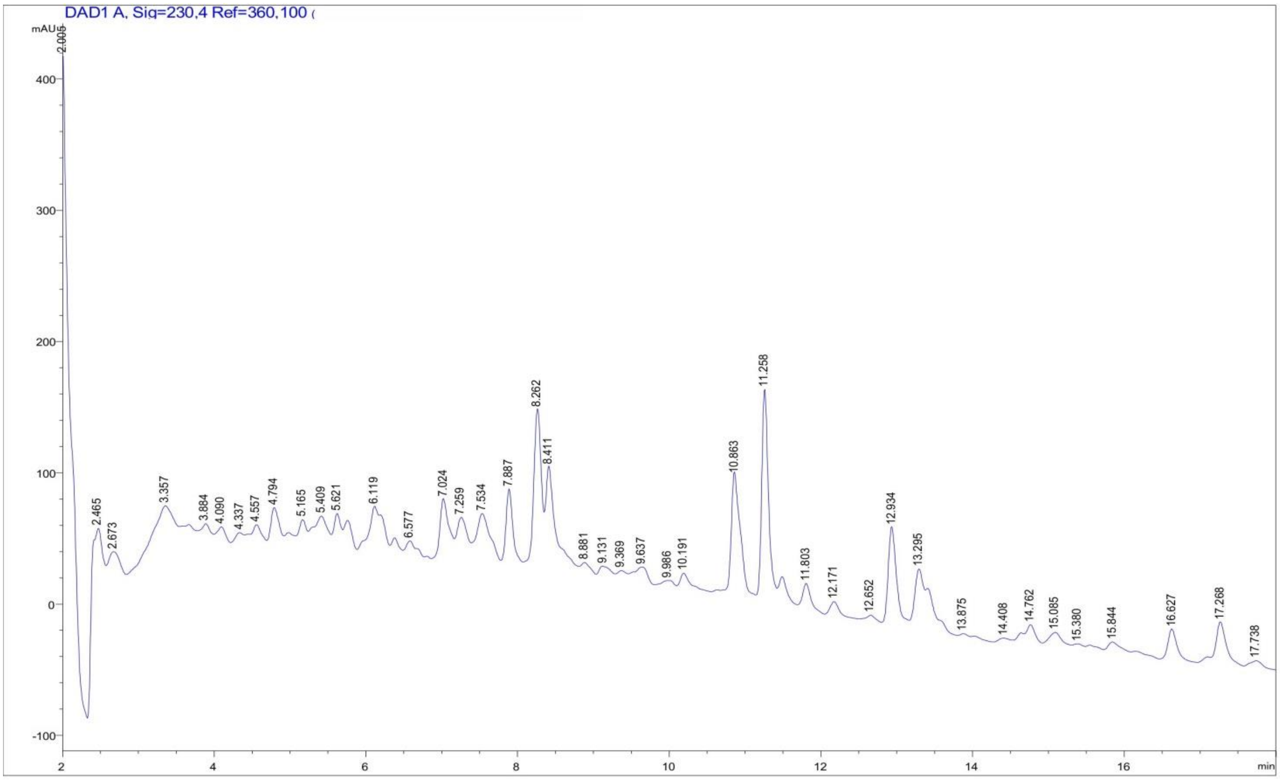

Figure S5. Chromatogram of *C. caeruleus* L. root aqueous extract at 230.4 nm

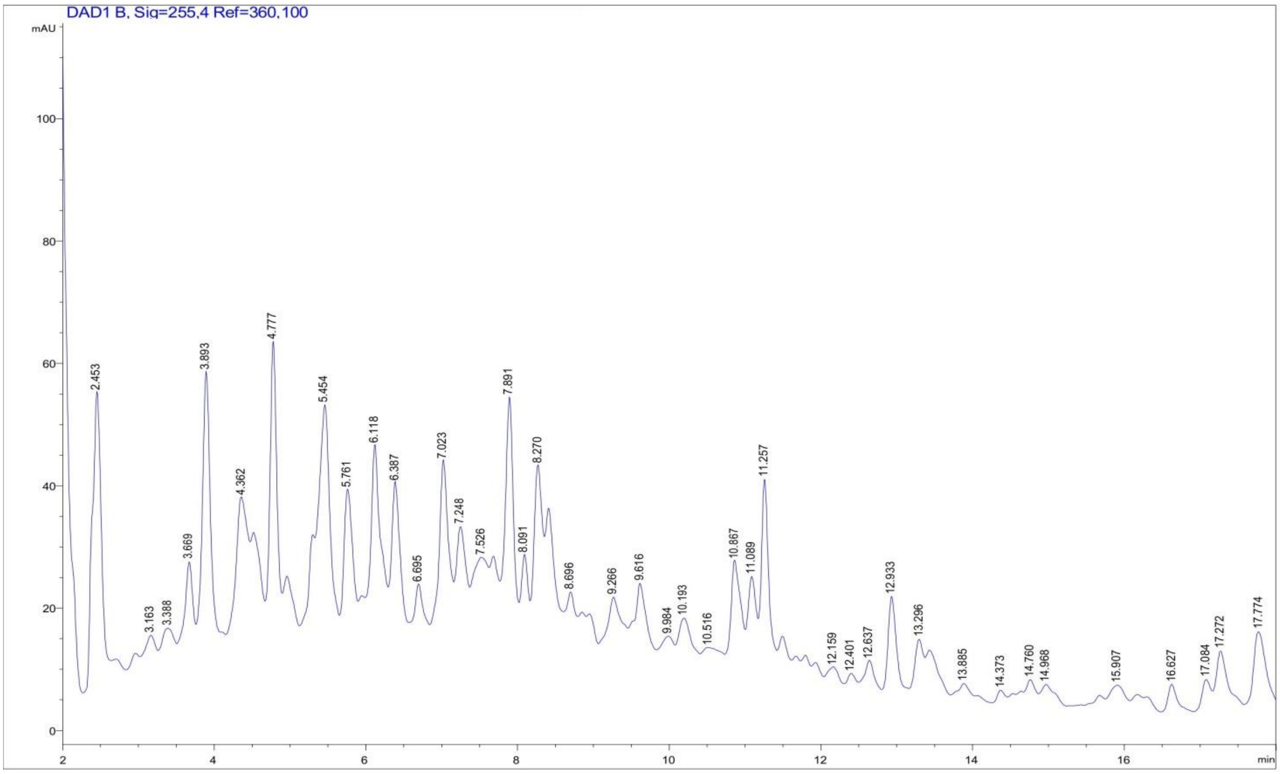

Figure S6. Chromatogram of *C. caeruleus* L. root aqueous extract at 255.4 nm

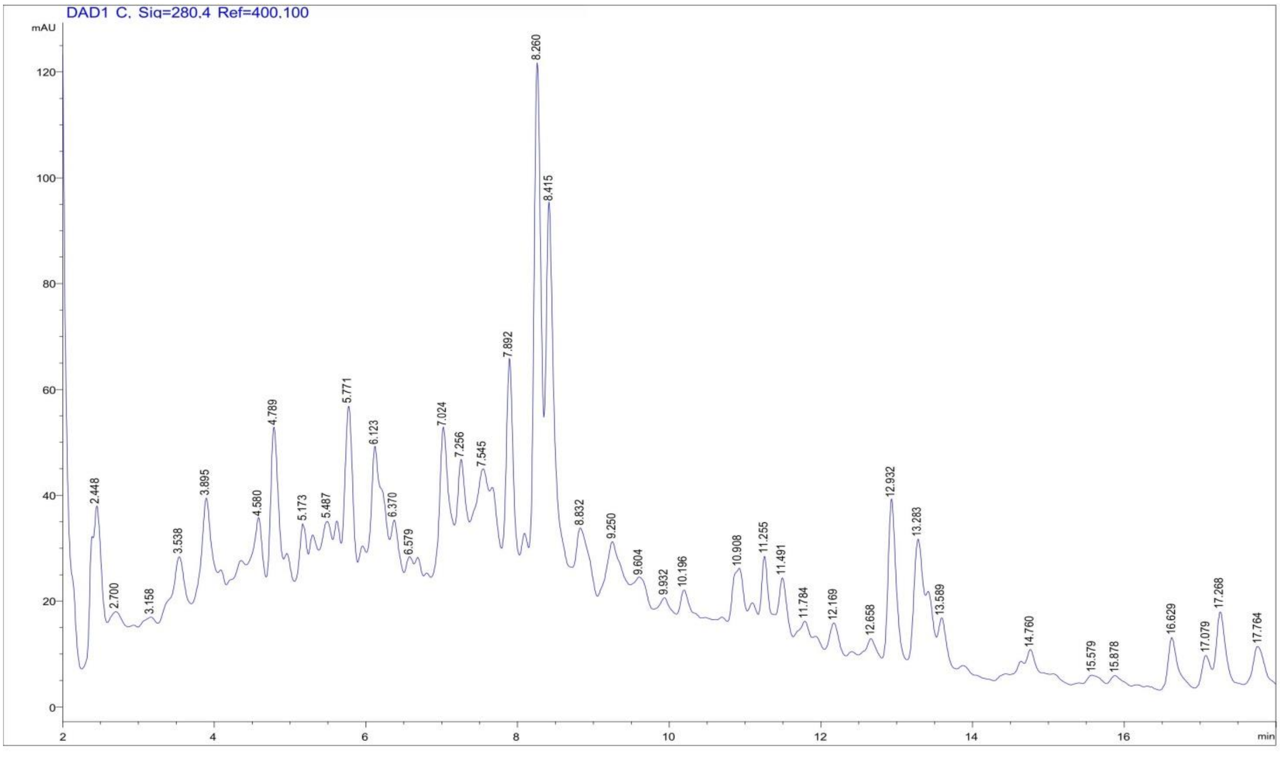

Figure S7. Chromatogram of *C. caeruleus* L. root aqueous extract at 280.4 nm

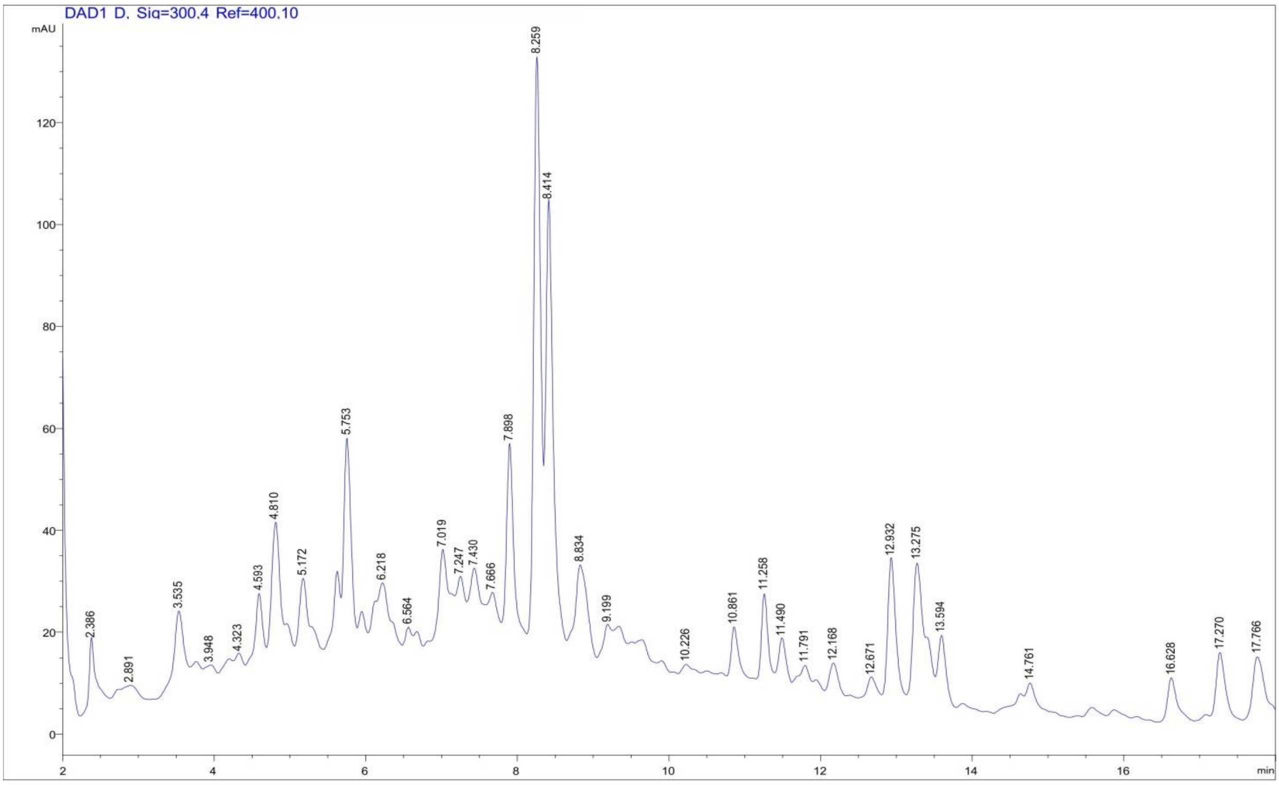

Figure S8. Chromatogram of *C. caeruleus* L. root aqueous extract at 300.4 nm

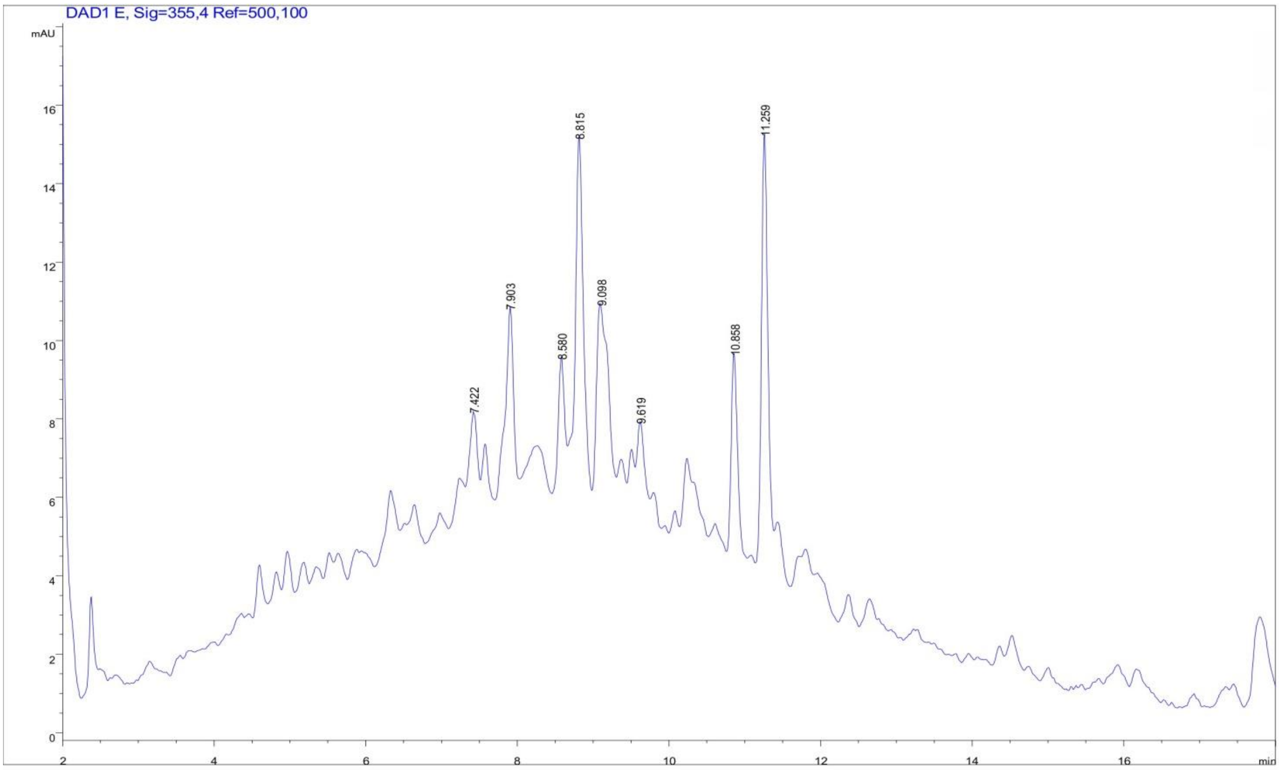

Figure S9. Chromatogram of *C. caeruleus* L. root aqueous extract at 355.4 nm

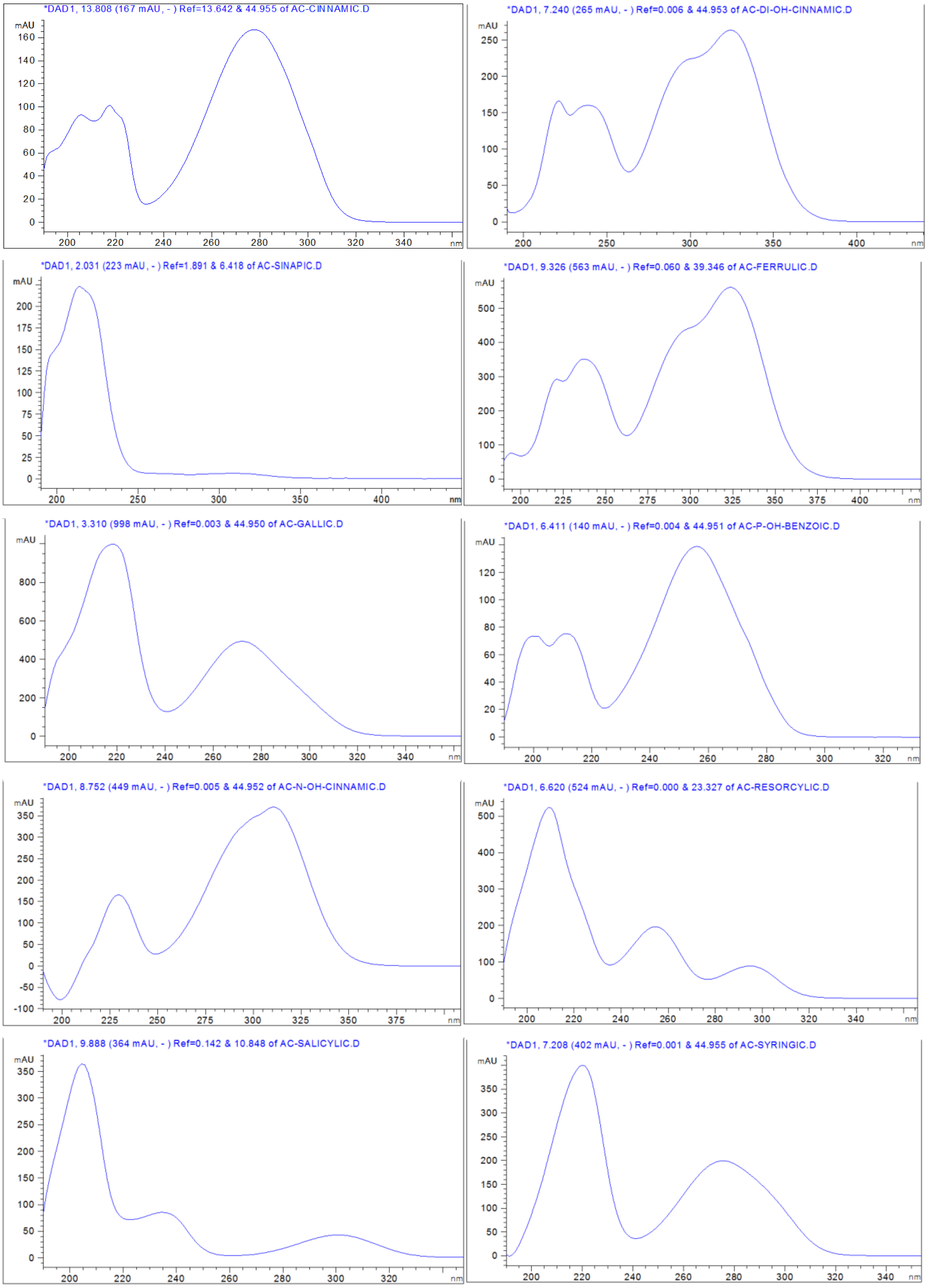

*DAD1, 13.808 (167 mAU, -) Ref=13.642 & 44.955 of AC-CINNAMIC.D
*DAD1, 7.240 (265 mAU, -) Ref=0.006 & 44.953 of AC-DI-OH-CINNAMIC.D
*DAD1, 2.031 (223 mAU, -) Ref=1.891 & 6.418 of AC-SINAPIC.D
*DAD1, 9.326 (563 mAU, -) Ref=0.060 & 39.346 of AC-FERRULIC.D
*DAD1, 3.310 (998 mAU, -) Ref=0.003 & 44.950 of AC-GALLIC.D
*DAD1, 6.411 (140 mAU, -) Ref=0.004 & 44.951 of AC-P-OH-BENZOIC.D
*DAD1, 8.752 (449 mAU, -) Ref=0.005 & 44.952 of AC-N-OH-CINNAMIC.D
*DAD1, 6.620 (524 mAU, -) Ref=0.000 & 23.327 of AC-RESORCYLIC.D
*DAD1, 9.888 (364 mAU, -) Ref=0.142 & 10.848 of AC-SALICYLIC.D
*DAD1, 7.208 (402 mAU, -) Ref=0.001 & 44.955 of AC-SYRINGIC.D
mAU
nm

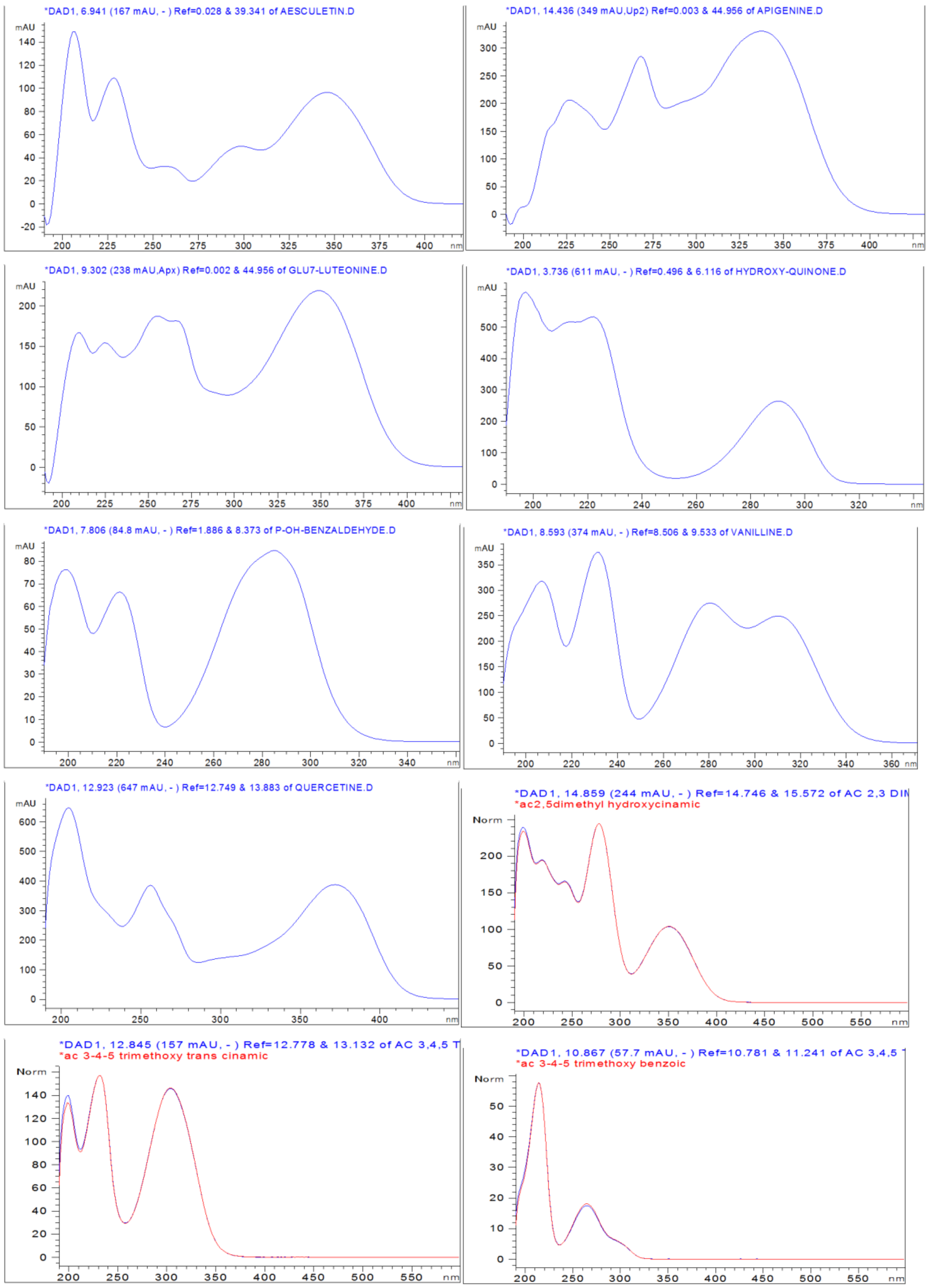
*DAD1, 6.941 (167 mAU, -) Ref=0.028 & 39.341 of AESCULETIN.D
*DAD1, 14.436 (349 mAU,Up2) Ref=0.003 & 44.956 of APIGENINE.D
*DAD1, 9.302 (238 mAU,Apx) Ref=0.002 & 44.956 of GLU7-LUTEONINE.D
*DAD1, 3.736 (611 mAU, -) Ref=0.496 & 6.116 of HYDROXY-QUINONE.D
*DAD1, 7.806 (84.8 mAU, -) Ref=1.886 & 8.373 of P-OH-BENZALDEHYDE.D
*DAD1, 8.593 (374 mAU, -) Ref=8.506 & 9.533 of VANILLINE.D
*DAD1, 12.923 (647 mAU, -) Ref=12.749 & 13.883 of QUERCETINE.D
*DAD1, 14.859 (244 mAU, -) Ref=14.746 & 15.572 of AC 2,3 DIM
*ac2,5dimethyl hydroxycinamic
*DAD1, 12.845 (157 mAU, -) Ref=12.778 & 13.132 of AC 3,4,5 T
*ac 3-4-5 trimethoxy trans cinamic
*DAD1, 10.867 (57.7 mAU, -) Ref=10.781 & 11.241 of AC 3,4,5 T
*ac 3-4-5 trimethoxy benzoic
mAU
Norm
nm

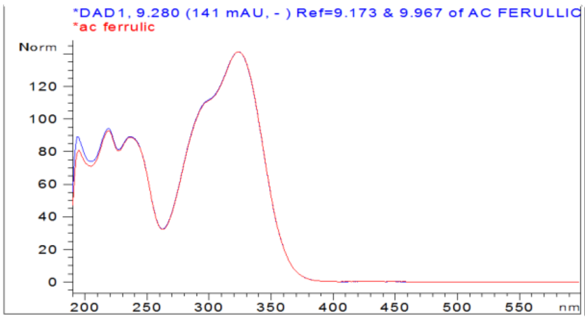
*DAD1, 9.280 (141 mAU, -) Ref=9.173 & 9.967 of AC FERULLIC
*ac ferrulic
Norm
0 20 40 60 80 100 120
200 250 300 350 400 450 500 550 nm

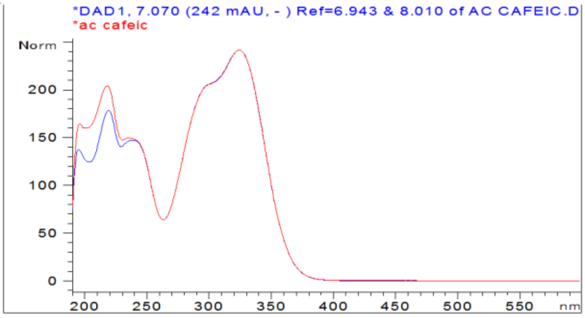
*DAD1, 7.070 (242 mAU, -) Ref=6.943 & 8.010 of AC CAFEIC.D
*ac cafeic
Norm
0 50 100 150 200
200 250 300 350 400 450 500 550 nm

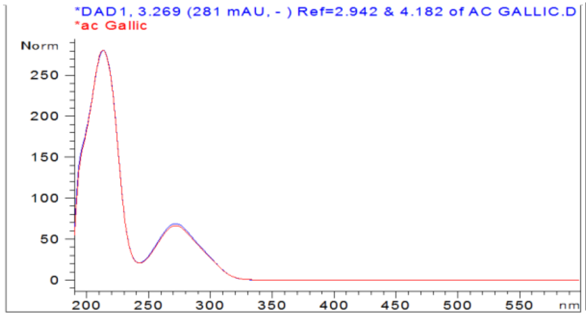
*DAD1, 3.269 (281 mAU, -) Ref=2.942 & 4.182 of AC GALLIC.D
*ac Gallic
Norm
0 50 100 150 200 250
200 250 300 350 400 450 500 550 nm

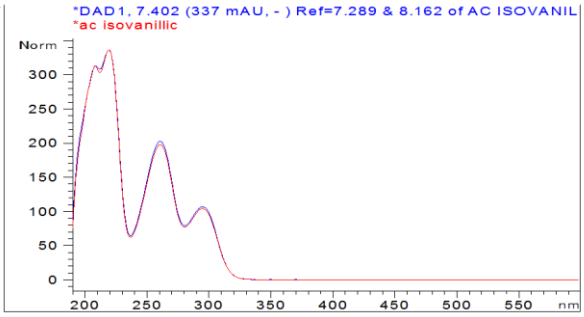
*DAD1, 7.402 (337 mAU, -) Ref=7.289 & 8.162 of AC ISOVANIL
*ac isovanillic
Norm
0 50 100 150 200 250 300
200 250 300 350 400 450 500 550 nm

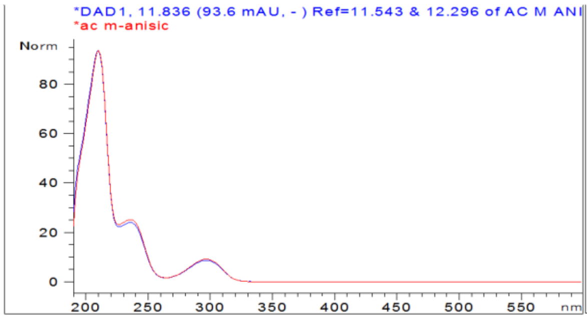
*DAD1, 11.836 (93.6 mAU, -) Ref=11.543 & 12.296 of AC M ANI
*ac m-anisic
Norm
0 20 40 60 80
200 250 300 350 400 450 500 550 nm

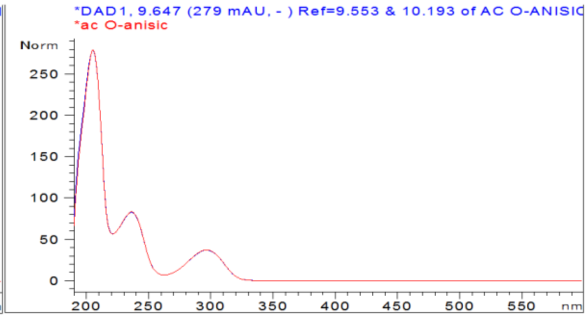
*DAD1, 9.647 (279 mAU, -) Ref=9.553 & 10.193 of AC O-ANISIC
*ac O-anisic
Norm
0 50 100 150 200 250
200 250 300 350 400 450 500 550 nm

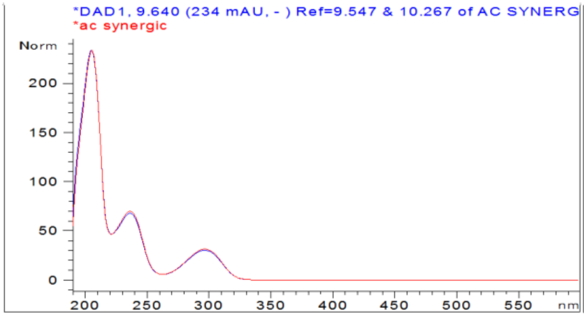
*DAD1, 9.640 (234 mAU, -) Ref=9.547 & 10.267 of AC SYNERG
*ac synergic
Norm
0 50 100 150 200
200 250 300 350 400 450 500 550 nm

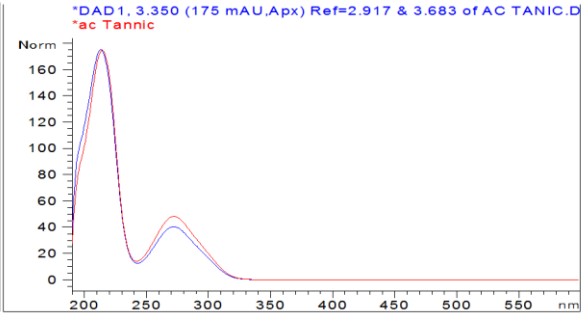
*DAD1, 3.350 (175 mAU,Apx) Ref=2.917 & 3.683 of AC TANIC.D
*ac Tannic
Norm
0 20 40 60 80 100 120 140 160
200 250 300 350 400 450 500 550 nm

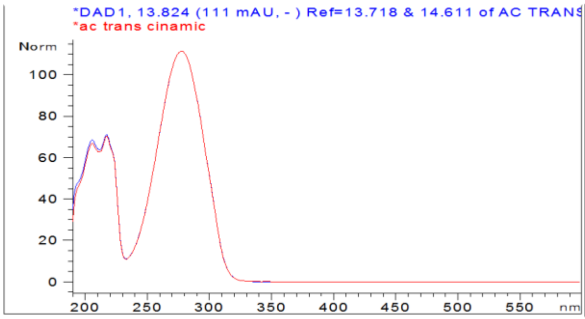
*DAD1, 13.824 (111 mAU, -) Ref=13.718 & 14.611 of AC TRANS
*ac trans cinamic
Norm
0 20 40 60 80 100
200 250 300 350 400 450 500 550 nm

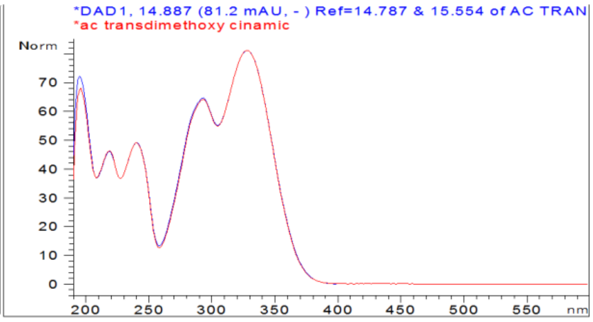
*DAD1, 14.887 (81.2 mAU, -) Ref=14.787 & 15.554 of AC TRAN
*ac transdimethoxy cinamic
Norm
0 10 20 30 40 50 60 70
200 250 300 350 400 450 500 550 nm

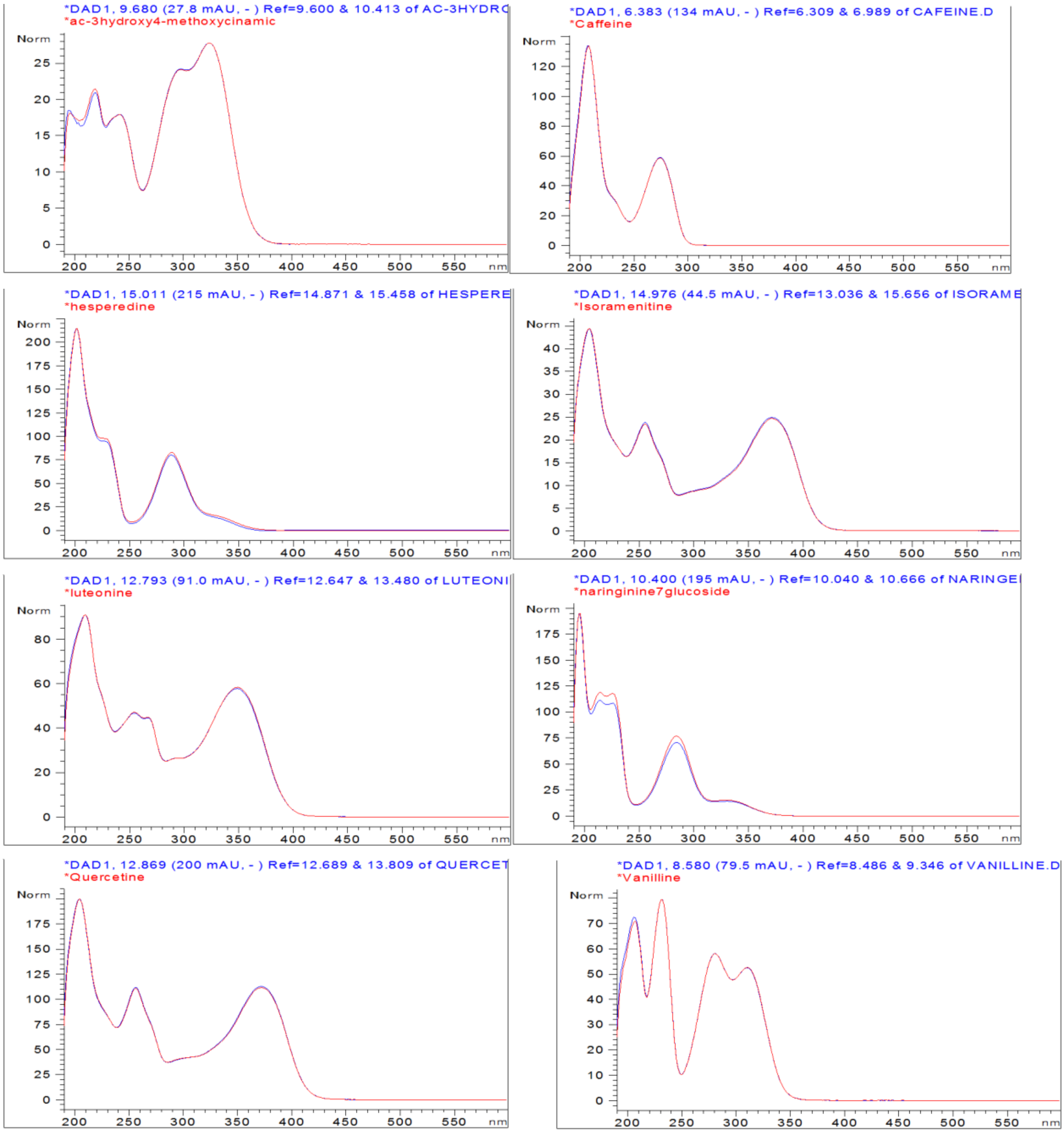

Figure S10. Spectrum of the mixture of reference standards
